# Statin Use and Coronary Artery Calcification: a Systematic Review and Meta-analysis of Observational Studies and Randomized Controlled Trials

**DOI:** 10.1007/s11883-023-01151-w

**Published:** 2023-10-05

**Authors:** Mitra Nekouei Shahraki, Soroush Mohammadi Jouabadi, Daniel Bos, Bruno H. Stricker, Fariba Ahmadizar

**Affiliations:** 1 Department of Epidemiology, Erasmus University Medical Center, Rotterdam, The Netherlands; 2 Division of Vascular Medicine and Pharmacology, Department of Internal Medicine, Erasmus University Medical Center, Rotterdam, The Netherlands; 3 Department of Radiology & Nuclear Medicine, Erasmus University Medical Center, Rotterdam, The Netherlands; 4 Department of Data Science and Biostatistics, Julius Global Health, University Medical Center Utrecht, Utrecht, The Netherlands

**Keywords:** Hydroxymethylglutaryl-CoA reductase inhibitors, HMG-CoA, Statins, Coronary vessels, Vascular calcification

## Abstract

**Purpose of Review:**

This review aimed to determine the association between statin use and coronary artery calcification (CAC), as detected by computed tomography in the general population, in previously published observational studies (OSs) and randomized controlled trials (RCTs).

**Recent Findings:**

A systematic search until February 2022 identified 41 relevant studies, comprising 29 OSs and 12 RCTs. We employed six meta-analysis models, stratifying studies based on design and effect metrics. For cohort studies, the pooled β of the association with CAC quantified by the Agatston score was 0.11 (95% *CI* = 0.05; 0.16), with an average follow-up time per person (AFTP) of 3.68 years. Cross-sectional studies indicated a pooled odds ratio of 2.11 (95% *CI* = 1.61; 2.78) for the presence of CAC. In RCTs, the pooled standardized mean differences (*SMD*s) for CAC, quantified by Agatston score or volume, over and AFTP of 1.25 years were not statistically significant (*SMD* =  − 0.06, 95% *CI* =  − 0.19; 0.06 and *SMD* = 0.26, 95% *CI* =  − 0.66; 1.19), but significantly different (*p*-value = 0.04). Meta-regression and subgroup analyses did not show any significant differences in pooled estimates across covariates.

**Summary:**

The effect of statins on CAC differs across study designs. OSs demonstrate associations between statin use and higher CAC scores and presence while being prone to confounding by indication. Effects from RCTs do not reach statistical significance and vary depending on the quantification method, hampering drawing conclusions. Further investigations are required to address the limitations inherent in each approach.

**Supplementary Information:**

The online version contains supplementary material available at 10.1007/s11883-023-01151-w.

## Introduction

Cardiovascular disease (CVD) is the primary cause of morbidity and mortality worldwide [1–3]. Coronary artery disease, in particular, is the primary cause of disability-adjusted life years lost globally [3–5], and its risk increases with age [6, 7]. Coronary artery calcification (CAC) is a dynamic and complex biological process linked to aging and serves as an index of arteriosclerosis [8–10]. Recent studies have shown that CAC is a predictor of CVD and major adverse cardiac events [11–13] leading to the endorsement of computed tomography (CT) CAC scoring for risk decisions at the primary prevention level [14, 15•]. Consequently, CAC has emerged as one of the indications for starting statin therapy, given its well-established cardiovascular protective effects. Statins are inhibitors of HMG-CoA reductase and are considered the most effective class of drugs for reducing low-density lipoprotein cholesterol (LDL-c) levels [16, 17]. However, recent studies on the pharmacological mechanisms of statins have suggested that they have the potential to accelerate vascular calcification, implying the possibility of a paradoxical effect [18].

The increase in vascular calcification by statins can be mainly attributed to their pleiotropic, LDL-independent, effects. The concept of statin pleiotropy emerged after fully accounting for statin’s clinical influence on CVD, and it received substantial pharmacological and molecular investigations [19, 20••]. Notably, statins can inhibit the synthesis of vitamin K2, a key cofactor for matrix Gla-protein in blood vessels, crucial for protecting against calcification [20••, 21–23]. Additionally, statins can suppress various macrophage phenotypes, promoting calcium deposition [23–25]. Interestingly, the burden of calcified atherosclerotic plaques has been found to correlate with the overall coronary plaque burden [18, 26]. However the effect of statins on CAC in the general population remained uncertain.

Several previous human subject studies have been conducted to investigate the association of statin use and CAC, with major variations in design and methodology. A wide range of evidence from observational studies has suggested that statin use increases CAC [27–32]. Some others, however, have concluded that statins reduce CAC [33–36]. Randomized controlled trials (RCTs) have also shown contradictory results [37–40]. Consequently, the genuine effect remains disputed and inconclusive [20••, 41]. There are some drawbacks that limited the previous systematic literature reviews and meta-analyses on this association [42–45]. They mostly did not thoroughly consider the profound diversity of included studies in terms of study design, outcome metrics, imaging modality, and quantification methods of CAC. Furthermore, most had search strategy constraints, such as limiting the publishing period to after a certain date, focusing on those reporting CVD events, or limiting the source population to specific comorbidities.

Despite these extensive researches on the relationship between statin use and CAC, the genuine effect of statins on CAC in the general population remains unclear. Therefore, to provide a more comprehensive understanding of prior research and to reduce sources of uncertainty and conflicting results, we aimed to conduct a systematic literature review and meta-analysis. Our objective is to investigate the association between statin use and CAC in the general population while accounting for variations in study design and methods.

## Methods

### Protocol, Search Strategy, and Selection Criteria

We developed a protocol submitted to PROSPERO on 10 June 2021 and registered with the number CRD42021254187. The selection process and reporting items were based on the preferred reporting items for systematic reviews and meta-analysis (PRISMA) flow diagram and checklist [46].

We formulated a comprehensive search strategy of Embase, Medline All via Ovid, Web of Science Core Collection, and Cochrane Central Register of Controlled Trials for publications without a language or time restriction up to 10 February 2022. A manual search to identify records through reference searches and gray, unpublished literature was conducted. Retrieved records were checked for inclusion and exclusion criteria after duplicates were removed. Observational studies and RCTs were eligible for inclusion if they investigated the association between statin use and CAC using a CT scan, the conventional imaging modality recommended for CAC scoring in clinical practice. The search strategy and inclusion and exclusion criteria were detailed in the supplementary material. In brief, any analytical observational study, comprising cross-sectional and longitudinal, that reported an effect estimate of the association between statin use and CAC was included. RCTs that measured CAC before and after the administration of statins were included. RCTs that only used statins in combination with non-statin medications in the same arm were excluded. The process was performed by two independent reviewers (M. N. S and S. M. J). Discrepancies over eligibility were resolved through consensus with a third reviewer (F. A.).

### Data Selection and Extraction

Two study investigators (M. N. S and S. M. J) independently extracted the data from the included records. The studies’ characteristics, including design, participants, exposure, outcome, and summary of statistical analysis were retrieved. From observational studies, effect sizes and corresponding 95% confidence intervals (*CI*) were collected. Where the 95% *CI* of the available effect estimate was not reported, we obtained it from the reported *p*-value [47]. We extracted the adjusted effect size, where both adjusted and unadjusted effects were reported.

The mean and the corresponding standard deviation (*SD*) or the median and the corresponding interquartile range (*IQR*) of CAC at baseline and follow-up were extracted from the included RCTs. Extracted median and *IQR* converted into *mean* and *SD* [48] for a unified outcome. Using that, we calculated the mean change from the baseline and its corresponding *SD* by a correlation coefficient for change from baseline [49] where it was not reported. CAC scores from the first and the last scans were extracted from studies with multiple follow-up scans (> 2 CTs). When RCTs undertook multiple arms, statin and placebo arms were used. If there were no placebo arms and only two arms with different statins or different dosages of statins were given, higher and lower defined daily doses (DDD) were identified according to the values of the World Health Organization; afterward, data of the lower DDD arm was used as the control arm.

### Quality and Risk of Bias Assessment

For quality and risk of bias assessment the Newcastle–Ottawa Scale [50] and Cochrane Risk of Bias assessment [51] tools were used.

### Statistical Analysis

#### Data Synthesis and Meta-analysis

Observational studies were classified first by their design and then by their outcome metric as continuous or binary. The effect size metrics and the corresponding 95% *CI* were unified in each group with odds ratios (*OR*) for binary outcomes (such as the prevalence of CAC), and beta coefficients for continuous outcomes (representing the CAC score). In RCTs, the mean change and corresponding *SD* of each study were used to measure the standardized mean difference (*SMD*), as the effect size. We conducted a meta-analysis when there were more than two studies that utilized the same study design, belonged to the same effect metric category, and, in the case of continuous outcomes, employed the identical CAC quantification method.

To pool the effect estimates, the inverse variance-weighted method was used. Using pre-calculated *OR*s, all were log-transformed before meta-analysis. *I*^2^ statistics were measured to quantify the variability in effect estimate due to between-study heterogeneity rather than chance. Meta-analysis of random effect models was generally applied. Exclusively, a fixed random effect model was applied only in the case of low (*I*^2^ < 25%) heterogeneity. Prediction interval (95% *PI*) was incorporated into random effect models to quantify the distribution of effect estimates and provides a range within which future research effects are anticipated to fall [52–54]. Influence diagnostic and leave-one-out analysis was conducted to detect the outliers and influential cases. If any outlier or influential case were detected, the meta-analysis model was once recalculated after removing the detected case. Visual assessment of the symmetry of the funnel plot and Egger’s test was done to identify small-study effect and publication bias. *P-*values were from 2-tailed tests, and if the *p*-value was < 0.05, the results were deemed statistically significant. All statistical analyses were performed using R studio for Windows V2021 and {meta}, {metaphor}, and {dmetar} packages [55–57].

#### Subgroup and Meta-regression Analysis

We used subgroup and meta-regression analyses to identify effect modifiers and other sources of heterogeneity. When there were at least eight studies, continuous factors were assessed in meta-regression models. Categorical variables were tested using subgroup analysis when there were at least ten studies in the meta-analysis model [55]. Subgroup analyses were performed based on the quantifying unit of CAC, DDD of the control arm, industry source of funding, follow-up time (long or short), quality assessment results (QAR). Each of the following variables was assessed in a separate meta-regression model: publication year, male proportion, average age, percentage of known CVD, mean baseline CAC score, follow-up time, and (QAR) (when continuous).

#### Synthesis Without Meta-analysis (SWiM)

Where including a study in a meta-analysis model was not possible, we used SWiM guidelines in systematic reviews [58], which occurred, for instance, when the description of the outcome of the association was too diverse from others to yield a meaningful summary estimate. We used arrows to visually summarize the direction of effect estimates for each study’s results. An indication of study size and statistical significance was used for the arrows, using size and color [58, 59].

#### Grading of Recommendations Assessment, Development, and Evaluation (GRADE)

The GRADE approach was used as a systematic and transparent judgment tool to assess the quality of the body of evidence for each outcome reported in the systematic review [60], as suggested by the Cochrane Collaboration [49]. The findings from the synthesis, with or without meta-analysis, were graded based on study design and seven other criteria. Downgrading factors included within-study risk of bias, imprecision of effect estimates, inconsistency, and indirectness. Upgrading factors included dose–response gradients, a large enough effect, and no plausible confounding or obvious bias. The GRADE method specifies quality into four levels: high, moderate, low, and very low.

## Results

### Search Results and Study Details

From 2377 initially identified records by the search, 188 full-texts were assessed for eligibility, leaving 41 original articles for inclusion in this systematic review. The PRISMA flow diagram of the selection process is available in the Supplementary material, Fig. S1. There were 13 cohort studies [27–29, 33, 34, 61–68], 16 cross-sectional studies [30, 32, 35, 36, 69–80], and 12 RCTs [37–40, 81–88] in this systematic literature review. This involved 12,520, 7072, and 1791 individuals, respectively. The average follow-up time per person was longer in cohorts compared to RCTs (3.90 years (*SD* = 1.27) and 1.25 years (*SD* = 0.25) respectively). The summarized features of included articles such as the study sample size, comorbidities, age, sex, the prevalence of CVD and statin use, follow-up time, and quantification method of CAC are presented in Tables 1, 2, and 3. In the last column of each table, the overall results of each study’s quality assessment are included. Details of the assessment are available in Tables S2–4.

**Table 1:** Characteristics of included cohort studies

	Source	Sample	Sample size, baseline	Known CVD%	Mean age, year	Men%	Statin use%	Follow-up, year	Imaging modality	Quantification method of CAC	Outcome	QAR^a^
Cohort studies, binary effect metric (*OR*)												
1	Raggi et al. [34] (the USA)	Indication for multiple CAC scanning	1310	0%	*n*/*a* [range: 30–80]	60%	37%	2.25	EBCT	Volume	Calcium score progression; either progression or not	7/9
2	Anand et al. [61] (the UK)	T2DM patients with a previous EBCT scan in the baseline	402	0%	52 (8)	56%	38%	2.5	EBCT	Volume	Calcium progression; either progression or not	7/9
3. 11	Chen et al. [62] (Sweden)	A cohort of ESRD patients	240	22%	56 [range: 29–77]	63%	16%	1.5	CT	AS	1-SD higher CAC score; either having or not	7/9
4. 14	Karpouzas et al. [63] (the USA)	Rheumatoid arthritis (RA)	101	0%	51.45 (10.29)	80%	40%	6.9	CTA	AS	CAC progression; either progression or not	9/9
5	Pechlivanis et al. [64] (Germany)	Randomly selected from the population for CAC scanning	3157	0%	59 (7.5)	47%	7.25%	5.1	EBCT	AS	CAC progression; either rapid or expected/slow	8/9
Cohort studies, continuous outcome metric (*β*)												
6	Shemesh et al. [65] (Israel)	Documented CAD with previous CAC scans	509	100%	65 (11)	87%	63%	4	MDCT	AS	Total calcification score after 4 years	8/9
7. 1	Hsia et al. [66] (the USA)	Healthy postmenopausal women with CAC score > 10	713	0%	65 (9)	0%	8%	3	CT	AS	Annual change in CAC	7/9
8. 2	Budoff et al. [33] (the USA)	T2DM patients with previous EBCT scans	163	0%	65 (10)	73%	49%	2.3	EBCT^1^	AS	Median annualized change in CAC score	7/9
9. 5	Elkeles et al. [29] (the UK)	The PREDICT cohort of T2DM patients	495	0%	*p*: 62.9 (6.9), *r*: 60.1 (6.7)	62%	13%	4	EBCT	AS	Change in CAC score	9/9
10. 6	Hoffmann et al. [67] (Germany)	Suspected for CAD with an indication for follow-up CAC scanning	63	~ 90%	63 (9)	71%	57%	2.08	CTA	Volume	Growth of calcified plaques	8/9
11. 7	Maréchal et al. [28] (Belgium)	Renal transplant cohort	300	~ 13%	52 (12)	29%	19%	3.5	CT	AS	Annualized change in CAC score	8/9
12. 8	Zeb et al. [68] (the USA	Indication for two consecutive CTA	100	0%	68.2 (8.2)	70%	60%	1.1	CTA	Volume	Change in CAC score	7/9
13. 15	Smit et al. [27] (five European countries)	Suspected for CAD with a previous CTA in the baseline	202	~ 100%	61 (9)	69%	80%	6.2	CTA	Volume	Annual change in CAC per patient	8/9

*CAC* coronary artery calcification, *Men%* percentage of men in the sample, *Statin use%* percentage of statin user in the sample, *QAR*^*a*^ quality assessment result, *OR* odds ratio, *β* beta coefficient, *CVD* cardiovascular disease, *T2DM* type 2 diabetes mellitus, *ESRD* end-stage renal disease, *CAD* coronary artery disease, *AS* Agatston score, *CT* computed tomography. *EBCT* electron beam computed tomography, *MDCT* multidetector computed tomography, *CTA* coronary computed tomography angiography, *n*/*a* not available, *p* progression group, *r* regression group

**Table 2:** Characteristics of included cross-sectional studies

	Source	Sample	*N*	Known CVD%	Age, mean (SD)/[range], yr	Men%	Statin use%	Imaging modality	Quantification method of CAC	Outcome	QAR^a^
Cross-sectional studies, binary effect metric (*OR*)											
	Jeon et al. [69] (South Korea)	Post-menopausal woman	436	0%	57.7 [range, 50 to 69]	0%	15%	MDCT	AS	CAC score > 100	8/9
	Hamer et al. [70] (the UK)	Healthy elderlies	443	0%	66 (6)	60%	22%	EBCT	AS	CAC score > 0	7/9
	Nakazato et al. [71] (six countries)	Previously underwent CCTA	6673	0%	59 (11)	55%	36%	CTA	Plaques that only contain calcification are described as calcified plaques	Presence of calcified plaque	8/9
	Greif et al. [72] (Germany)	Previously underwent CAC scan	1560	100%	m: 55.4 (19), f: 63.2 (22)	72%	*n*/a*	CT scan	AS	CAC score > 0	7/9
	Shikada et al. [73] (Japan)	Suspected of IHD	201	7%	64 [range, > 20]	65%	40%	CT scan	AS	CAC score > 238	7/9
	Panh et al. [74] (France)	Referred for CVD risk stratification	500	0%	60.9 (10.8)	50%	37%	CT scan	AS	CAC score > 0	7/9
	Lee et al. [75] (South Korea)	Suspected of metabolic syndrome	847	0%	56.7 (6.57)	50%	23.8%	CT scan	AS	CAC score > 0	9/9
	Drouin-Chartier et al. [76] (Canada)	Genetically defined HeFH	146	12%	47.8 (14.1)	53%	97%	CT scan	AS	CAC score > 0	9/9
	Béland-Bonenfant et al. [77] (Canada)	Genetically defined HeFH	62	0%	48 (14)	48%	100%	CT scan	AS	CAC score > 0	6/9
Cross-sectional studies, continuous outcome metric (*β*)											
	Elkeles et al. [78] (the UK)	T2DM	495	0%	62.9 (7.1)	67%	36%	EBCT	AS	CAC score	8/9
	Nguyen et al. [79] (Belgium)	Renal transplant recipients	281	0%	53 (13)	61%	40%	CT	Calcium mass score	Calcium mass score	8/9
	Hosseinsabet et al. [36] (Iran)	Admitted for CABG	143	100%	57.7 (9.4)	74%	62%	EBCT	AS	CAC score	7/9
	Cheng et al. [32] (the USA)	Newly diagnosed CAD	823	100%	65 (12)	64%	52%	CTA	Plaques with more than 75% calcification in their volume are described as calcified plaques	Absolute calcified plaque count	7/9
	Jung et al. [35] (South Korea)	T2DM	110	0%	57 (11)	65%	59%	MDCT	AS	CAC score	5/9
	Rodriguez et al. [30] (the USA)	Asymptomatic statin-eligible adults	199	0%	65.5 (6.9)	64%	100%	CT scan	AS	CAC score	7/9
	Zhelyazkova-Savova [80] (Bulgaria)	Moderate-to-high risk for CVD	98	100%	62.12 (12)	35%	31.6%	MSCT scan	AS	CAC score	5/9

*CAC* coronary artery calcification, *Men%* percentage of men in the sample, *Statin use%* percentage of statin user in the sample, *QAR*^*a*^ quality assessment result, *OR* odds ratio, *β* beta coefficient, *CVD* cardiovascular disease, *T2DM* type 2 diabetes mellitus, *ESRD* end, stage renal disease, *IHD* sschemic heart disease, *CCTA* coronary computed tomography angiography, *HeFH* heterozygous familial hypercholesterolemia, *CABG* coronary artery bypass graft, *CAD* coronary artery disease, *AS* Agatston score, *CT* computed tomography. *EBCT* electron beam computed tomography, *MDCT* multidetector computed tomography, *MSCT* multi-slice computed tomography, *CTA* coronary computed tomography angiography. *n*/*a* not available

**Table 3:** Characteristics of included randomized controlled trials (RCTs)

	Source	Allocation	Known CVD%		Comorbidities	Age at baseline, mean (SD), [range], year	Men%	CT	Arm 1: comparator (*N*)	Arm 2: intervention (*N*)	Follow-up, year	Quantifying unit	Industrial source of founding	QAR^a^
RCTs with a placebo or no-treatment control group														
1	Houslay et al. [81] (the UK)	Randomized, open-label	28%		Calcific aortic stenosis	a: 70 (8), b: 70 (9)	76%	H-CT	Placebo (78)	Atorvastatin 80 mg/day (77)	2	AS	Yes	L
2	Terry et al. [40] (the USA)	Randomized, single-blind	0%		Asymptomatic for CVD, with dyslipidemia and CAC AS > 50	a: 66 (6), b: 66 (5) [range, 21–5]	91%	MDCT	Placebo (40)	Simvastatin 80 mg (40)	1	AS, volume	Yes	L
3	Dichtl et al. [37] (Austria)	Randomized	0%		Asymptomatic calcified aortic stenosis	a: 67 (10.6), b: 64.2 (12) [range, > 18]	59%	MDCT	Placebo (16)	Atorvastatin 20 mg (19)	2	AS	No	H
4	Petri et al. [82] (the USA)	Randomized, single-blind		0%	Clinically diagnosed SLE	44.7 (11.3), [range, 18–78]	9%	H-CT	Placebo (91)	Atorvastatin 40 mg (96)	2	AS, volume	No	H
5	Plazak et al. [38] (the USA)	Randomized, double-blind		0%	Treated for systematic SLE	41.8 [range, 20–73]	10%	MDCT	Placebo (32)	Atorvastatin 40 mg/day (28)	1	AS	No	L
6	Lemos et al. [83] (Brazil)	Randomized, open-label		Not assessed	Chronic kidney disease	57 (11)	62%	MSCT	Controls (41)	Rosuvastatin 10 mg/day (38)	2	AS	No	U
7	Lo et al. [84] (the USA)	Randomized, double-blind		0%	HIV-positive patients on stable antiretroviral therapy	51 (5)	80%	CTA	Placebo (20)	Atorvastatin 20 mg/day escalated to 40 mg/day at the 3-month visit (17)	1	AS, volume	No	L
8	Yazbek et al. [85] (Brazil)	Randomized, open-label		0%	Kidney transplant recipients	41 (10)	56%	MSCT	Control (59)	Atorvastatin 10 mg/d (61)	1	AS	No	L
RCTs with a low-dose-statin control group														
9	Raggi et al. [86] (the USA)	Randomized, double-blind		25%	Menopausal women with CAC volume > 30 mm	64 (6) [range, 55–75]	0%	EBCT	Atorvastatin 80 mg/day (218)	Pravastatin 40 mg/day (257)	1	Volume	Yes	L
10	Schmermund et al. [87] (three countries^b^)	Randomized, double-blind		0%	More than two CVD risk factors	61 (8)	74%	EBCT	Atorvastatin 10 mg/day (191)	Atorvastatin 80 mg/day (175)	1	AS, volume	Yes	L
11	Auscher et al. [88] (Denmark)	Randomized, open-label		100%	Acute myocardial infarction	62, [range,53–63]	37%	CTA	Simvastatin 40 mg/day (48)	Rosuvastatin 40 mg/day (48)	1	Volume	No	U
12	Miyoshi et al. [39] (Japan)	Randomized, open-label		0%	Hyperlipidemia, CAC AS > 0	66 (9), [range, 20-]	55%	MDCT	Pitavastatin 2 mg/day (55)	Pitavastatin 4 mg/day (46)	1	AS, volume	Yes	U

*CAC* coronary artery calcification, *N* number of subjects in the bassline, *Men%* percentage of men in the sample, *QAR*^*a*^ quality assessment results, overall risk of bias, *AS* Agatston score, *CVD* cardiovascular disease, *SLE* systemic lupus erythematosus, *HIV* human immunodeficiency virus, *CT* computed tomography, EBCT electron beam computed tomography. *MDCT* multi-detector computed tomography. *MSCT* multi-slice computed tomography. *H-CT* helical computed tomography. *CTA* coronary computed tomography angiography, *H* high risk of bias, *L* low risk of bias, *U* unclear

^a^Comparator comparator arm

^b^Statin use arm, three countries: Germany, England, and Russia

### Meta-analyses

Six meta-analysis models were developed by using 32 studies (Figs. S2–7). Table 4 provides data on the pooled results before and after removing outliers and influential cases. Across the six models, two showed statistically significant associations. One of which is the model of cohort studies which showed statin use was significantly associated with CAC — quantified as Agatston score — (B-coefficient: 0.11, 95% *CI*: 0.05; 0.16). The model showed no heterogeneity (*I*^2^: 0.0%), and the included cohorts had an average follow-up per person of 3.68 years. The other model is the model of cross-sectional studies. The pooled odds of the presence of CAC in those who used vs did not use statins was 2.11 (95% *CI*: 1.61; 2.78). This model had high heterogeneity (*I*^2^: 69.7%), and a statistically significant prediction interval (95% *PI*: 1.00; 4.53). The available data were insufficient to standardize the beta coefficients before the meta-analysis. In RCTs, the pooled effect estimates with CAC quantification in Agatston score or volume were not statistically significant (*SMD*: − 0.06, 95% *CI*: − 0.19; 0.06, *I*^2^ = 0.0% and *SMD*: 0.26, 95% *CI*: − 0.66; 1.19, *I*^2^ = 77.6%; Table 4).

**Table 4:** Pooled effect size estimate of the six meta-analysis models before and after removing the outliers and influential cases

Model, quantifying unit of CAC, (outcome)		*N*	Pooled effect size	95% *CI*, 95% *PI*	*I*^2^
1	Meta-analysis of cohort studies with binary effect metric (progression of CAC)	4	*OR* = 1.10	[0.57; 2.12], [0.05; 26.20]	93.5%
2	Meta-analysis of cohort studies with continuous effect metric (Agatston score)	4	*Beta* = 0.11	[0.05; 0.16], [− 0.02; 0.23]	55.8%
	Outliers and influential cases removed^a^	3	*Beta* = 0.11	[ 0.05; 0.16]	0.0%
3	Meta-analysis of cross-sectional studies with binary effect metric (prevalence of CAC)	8	*OR* = 1.78	[1.12; 2.83], [0.41; 7.80]	77.1%
	Outliers and influential cases removed^b^	7	*OR* = 2.11	[1.61; 2.78] [1.0; 4.53]	69.7%
4	Meta-analysis of cross-sectional studies with continuous effect metric (Agatston score)	5	*Beta* = 0.04	[− 0.24; 0.31], [− 0.95; 1.02]	90.1%
5	Main analysis of RCTs quantified CAC as Agatston score	10	*SMD* = − 0.07	[− 0.38; 0.24], [− 0.95; 0.81]	73.6%
	Outliers and influential cases removed^c^	8	*SMD* = − 0.06	[− 0.19; 0.06]	0.0%
6	Main analysis of RCTs quantified CAC score as volume	6	*SMD* = 0.26	[− 0.11; 0.63], [− 0.66; 1.16]	77.6%

*N* number of included studies, *CI* confidence interval, *PI* prediction interval of the random effect, *I*^2^ variability in effect estimate due to between-study heterogeneity, *CAC* coronary artery calcification, *RCTs* randomized controlled trials. *OR* odds ratio, beta beta-coefficient, *SMD* standard mean difference

^a^Removed as an outlier: “Hsia et al.”

^b^Removed as an outlier: “Panh et al.”

^c^Removed as outliers: “Plazak et al.”, “Miyoshi et al.”

### Subgroup and Meta-regression Analysis

The subgroup analyses showed a statistically significant difference in the pooled effect size based on whether CAC was quantified as Agatston score or volume (Table 5; 95% *CI*: [0.02 to 0.62]). Detailed results of other subgroup analyses are presented in Tables S6–9.

**Table 5:** Subgroup analysis of randomized controlled trials (RCTs) based on the quantifying unit of CAC as either Agatston score or volume

Model, quantifying unit of CAC		*N*	Pooled effect size	95% *CI*, 95% *PI*	*I*^2^	95% *CI*
1	Meta-analysis of RCTs, Agatston score	10	*SMD* = − 0.07	[− 0.38; 0.24], [− 0.95; 0.81]	73.6%	[0.02; 0.62]*
	Outliers and influential cases removed^a^	8	*SMD* = − 0.06	[− 0.19; 0.06]	0.0%	
2	Meta-analysis of RCTs, volume	6	*SMD* = 0.26	[− 0.11; 0.63], [− 0.66; 1.16]	77.6%	

*N* number of included studies, *CI* confidence interval, *PI* prediction interval of the random effect, *CAC* coronary artery calcification, *I*^2^ variability in effect estimate due to between-study heterogeneity, *SMD* standard mean difference

* Unpaired *t*-test of the pooled effect estimates, after removing outliers and influential cases. The two-tailed *p* value equals 0.037, and the difference is considered to be statistically significant

^a^Removed as outliers: “Plazak et al.,” “Miyoshi et al.”

Meta-regression was conducted in two meta-analysis models, the model of cross-sectional studies with binary effect metrics and the model of RCTs which quantified CAC as Agatston score. Table 6 displays the estimated effect for each variable in meta-regression and the difference in the true effect size explained by each variable. None of the included variables had a statistically significant modification in the estimated effect.

**Table 6:** Meta-regression of the meta-analysis models

Model, quantifying unit of CAC (*N*)	Variable^a^	Estimated regression coefficients^b^	95% *CI*	*R*^2c^
Meta-analysis of RCTs, Agatston score (*N* = 10)	Publication year	0.03	− 0.04, 0.10	0.00%
	Male%	0.50	− 0.70, 1.70	0.00%
	Mean age	0.02	− 0.01, 0.04	5.40%
	CVD%	0.34	− 3.45, 4.14	0.00%
	Follow-up time	− 0.06	− 0.69, 0.57	0.00%
	Mean CAC score at the baseline	− 0.001	− 0.01, 0.01	0.00%
Meta-analysis of cross-sectional studies with binary effect metric (*N* = 8)	Publication year	− 0.13	− 0.28, 0.03	10.07%
	Male%	0.23	− 1.60, 2.06	0.00%
	Mean age	0.05	− 0.05, 0.15	0.00%
	CVD%	0.42	− 0.63, 1.48	0.00%
	QAS	− 0.06	− 0.66, 0.54	0.00%

Meta-regression was conducted in the models with at least eight included studies

*N* number of included studies, *CI* confidence interval of the effect, *R*^2*c*^ the difference in the true effect size that can be explained by the potential effect modifier, *RCT* randomized control trials, *CAC* coronary artery calcification, *male%* percentage of males in the study population, *CVD%* percentage of cardiovascular disease in the study population, *QAS* quality assessment results of observational studies in a continuous format

^a^Potential effect modifier

^b^Effect estimate of each variable

### Small-Study Effect and Publication Bias

Visual inspection of the funnel plots and the Eggers’ regression test results indicate small-study effects in the meta-analysis of RCTs quantified CAC in volume (Egger’s intercept: 3.78, 95% *CI*: 1.41; 6.16). The contour-enhanced funnel plot shows that the available small studies are more likely to have a larger effect size and a more significant result when compared to the larger available studies. The funnel plots and the details of the Egger regression tests are available in Figs. S14–20 and Table S10, respectively.

#### SWiM

Inclusion in the meta-analysis was precluded for nine observational studies. In one cross-sectional study with a binary outcome, statin use was significantly associated with the CAC score > 238 [73]. In the two other studies with a continuous outcome, statin use was significantly associated with rising calcium mass scores and absolute calcified plaque counts [31, 32]. One cohort study with a dichotomous outcome revealed that statin use was significantly associated with a rapid progression of CAC [64]. One out of the four studies with a continuous outcome showed statin use was significantly associated with a decrease in the median CAC progression [33]; the other three identified that statin use was associated with an increase in CAC [27, 67, 68], with only one reaching statistical significance. Tables S11–12 provide SWiM-detailed results accompanied by the reasons for exclusion from meta-analysis, according to McKenzie and Brennan [89].

#### GRADE

The confidence level of the body of evidence in RCT meta-analyses was high, while the confidence levels in observational studies, both with and without meta-analysis, were rated as low or very low. Detailed results of the GRADE approach are available in Table S13.

## Discussion

The present study summarized quantitative evidence from previously published observational studies and RCTs on the association between statin use and CAC detected by CT scans in the general population. In observational studies, two meta-analysis models showed significant associations: one between statin use and increasing CAC, as quantified in the Agatston score, and the other with the presence of CAC. These results were inferred from cohort studies with an average follow-up time per person of 3.68 years and cross-sectional studies, respectively. Meta-analyses of RCTs did not reach statistical significance; however, the pooled effects differed significantly depending on whether CAC was quantified as Agatston score or volume.

Our findings from observational studies are supported by the results from in vivo investigations of the effect of statins on vascular calcification, which suggest an increase in calcification. These findings, potentially, contradict the expected cardiovascular protective effects of statins on CAC. Some explanations for this effect propose that statins may stabilize atherosclerotic plaques and prevent plaque rupture by increasing calcification. It is important to note that the beneficial effects of statins on coronary atherosclerotic plaque primarily manifest through their ability to slow down or reverse the progression of soft plaque components. Soft plaque components including lipid-rich core are particularly dangerous because such plaques are unstable and vulnerable to rupture [18, 90–92]. However, we would like to highlight that our findings from meta-analyses of observational studies were influenced by the inherent limitations of observational association studies which employed descriptive or etiological approaches. Among the most important ones is confounding and, in the case of our study, confounding by indication, which compromises the reliability of the conclusions regarding the genuine effect of statins. These inherent limitations also resulted in low or very low rates in the GRADE system used to assess the quality of evidence. As a result, it is uncertain how much of the observed effect estimate, suggesting an increase in CAC, would remain after accounting for confounding related to statin indication. Interestingly, the results from the two meta-analysis models of observational studies with statistically significant findings suggest a consistent pattern of association across included studies. This implication is supported by the use of a fixed-effect meta-analysis for cohort studies and the significant prediction intervals obtained from the random-effect meta-analysis for cross-sectional studies, after excluding the outliers. Therefore, there is a high probability that future observational studies employing similar designs and outcome metrics will yield similar effect estimates, showing an increase in CAC, in this level of multivariable-adjusted associations [53, 54]. Future observational studies are needed to address confounding using innovative causal inference methods. These methods can estimate causal effects without the need to measure all confounding factors, allowing for conclusions regarding the drug’s effects in the general population.

Our findings from RCTs in both quantification methods did not reach conventional levels of statistical significance after an average of 1.25-year period. The findings have a high-quality score, as indicated by the GRADE approach. They are also clinically relevant and promissings since an increased amount of CAC over serial scans has been closely associated with a higher risk of future cardiovascular events [11–13]. However, we believe that with this quite solid and yet limited available evidence obtained from RCTs, we cannot yet draw a comprehensive conclusion about the full scope of statin’s effect on CAC, since the drug’s effect on the CAC could be dynamic over time. Although the effect modification of follow-up time on the results from RCTs did not reach the statistical significance in both subgroup and meta-regression analysis, both analyses showed a trend toward smaller effect sizes as the follow-up was prolonged (range of 1–2 years) which aligns with our expectations. The pooled effects in cohorts and RCTs were assessed at the point in their follow-up period after the effect was detectable in clinical settings. This is inferred from statins’ in-clinic response time ranging from 4 to 12 weeks [93]. If the effect size were to continue increasing indefinitely, CAC would eventually reach a point where it obstructs blood flow, contradicting the established cardiovascular protective effect of statins. As a result, we expect the estimated effect size of statins on CAC to decline over time. This dilution of effect could possibly be affected in part by the natural aging-related increase in CAC. We think that a more significant effect size may be observed by assessing CAC immediately after and near the in-clinic response time. Considering the limited duration of time and degrees of freedom supplied by our data, which prevents drawing furthur conclusions about the effect in short-term vs long-term follow-up, future studies with tailored follow-up time customized to the pharmacological properties of statins are warranted.

The model of RTCs in volumetric units, one of our six meta-analysis models, was influenced by the small-study effect, which may indicate publication bias. This is despite our efforts to reduce the likelihood of this bias by undertaking the reference search and covering the gray and unpublished literature in our manual search. Since we observed that the small studies were more prone to publication bias and were more likely to have significant results, we think the missing studies are small studies with insignificant results. As a result, outcome reporting bias is probable, provided that RCTs mainly measure multiple outcomes of interest. It is likely that insignificant results in volumetric units, a less common method of quantification in clinics, were dropped.

The RCTs in this meta-analysis allowed us to compare the pooled effect estimates across the two quantification methods. Although the Agatston score and volume remain very closely correlated [94], the significant difference between the pooled effects could be attributed to what each scoring technique measures and the limitations inherent in each technique. The most commonly used method is the Agatston score, which is calculated by multiplying the total area of voxels (mm^2^) by an arbitrary density index based on the voxel with the highest density, ranging from one to four [94–96]. As a result, it is affected simultaneously by calcification area and density, and due to the weighted density index, it grows non-linearly as density increases. CAC volume score provides an alternate method of CAC scoring. It is calculated by multiplying the total number of calcium-containing voxels by the volume of 1 voxel [94, 95]. Volume score is considered more relevant for assessing CAC evolution over time since it allows for a linear augmentation when calcium rises [94, 95, 97]. By contrasting the pooled effect estimates between the two quantification techniques, we have highlighted the distinct potential effect of statin use on the density, area, and volume of CAC.

Recent data suggests that increased density in calcified coronary plaques could be protective against coronary artery diseases and major adverse events, consistent with the concept that it may increase the stability of atherosclerotic plaques and reduce the risk of plaque rupture [41, 98••]. The Agatston score, which was developed without specific histopathological data for correlation, assumes, by definition, that high-density CAC plaque is associated with a higher incidence of coronary artery diseases [94]. Recent evidence using coronary CT angiography and intravascular ultrasound (IVUS) has supported statin’s effect predominantly on increasing dense calcium [98••, 99]. Although the Agatston score is an independent predictor of cardiovascular risk, we hypothesize that by distinctly adding CAC density, area, and volume, the predictive value might be improved since the impact of statins on these measures could be different. Additionally, we believe there may be added value in using the inverse relation between CAC density and volume. This may help quantify what is already suggested as the stability and maturity of calcium within an atherosclerotic plaque.

### Limitations and Strengths

We studied drug use as the exposure of the association under investigation; therefore, the duration and intensity of the exposure were not covered by the pooled results. Studying the general population, we designed to investigate the effect of the population’s risk profile on the association, which showed no modification in the effect. However, since few records were included in some meta-analysis models, we could not run subgroup or meta-regression analyses across all the models. We had limited data to provide a standardized measure of the beta coefficients in the analyses. However, it may not change the overall trend of the results considerably, given that the outcome data in most studies underwent the same transformation. Our meta-analysis employed study-level data, not individual patient-level data, which could have assisted in overcoming some of the already-mentioned limitations of this study.

Our study has a number of strengths. Statins have received the most extensive research attention in cardiovascular pharmacology. Our findings, building upon previous results, provided new insight into our understanding of the effect of these medicinal products on CAC in the general population. We assessed our findings’ robustness, inspected heterogeneity patterns, and discussed reasons why the effects differed. These approaches helped us develop informed hypotheses and conclusions from observational studies and RCTs in our systematic review and meta-analysis.

## Conclusion

Drawing from our findings, the effects of statins on CAC demonstrate variations across different study designs and effect size metrics. Cohort and cross-sectional studies suggest a significant association between statin use and CAC score progression and CAC presence, respectively. Nevertheless, RCTs did not determine a significant effect, with the effects being different across the quantification unit of CAC, hindering forming a conclusion. Interpretation should consider limitations inherent in included studies, namely confounding by indication in observational studies, variations in CAC quantification method, and limited follow-up time points.

To gain a better understanding of this association, a large long-term RCT is required to consider the effect over customized follow-up times aligned with the pharmacological properties of statins. However, ethical, medical, and logistical aspects may restrict the feasibility of it. Observational studies with large sample sizes drawn from the general population could shed light on this association by taking one step towards addressing confounding by indication and establishing causality. Furthermore, future studies should independently assess the volume and density of the observed effects, thus providing a more detailed characterization of the effect.

### Supplementary Information

Below is the link to the electronic supplementary material.

Supplementary file1 (DOCX 5.34 MB)

## Data Availability

The data are available upon reasonable requests made to the corresponding author.
