# Supplementary material for: Statin Use and Coronary Artery Calcification: a Systematic Review and Meta-analysis of Observational Studies and Randomized Controlled Trials
Source: Curr Atheroscler Rep. 2023 Oct 5;25(11):769–84. doi: 10.1007/s11883-023-01151-w (PMC10618336; doi:10.1007/s11883-023-01151-w)
Supplement: Supplementary file 1 — Supplementary file1 (DOCX 5.34 MB) [file 11883_2023_1151_MOESM1_ESM.docx]

**Supplementary Information**

**Statin use and coronary artery calcification: A systematic review and meta-analysis of observational studies and randomized controlled trials**

Mitra Nekouei Shahraki, M.D, M.Sc^a^, Soroush Mohammadi Jouabadi, Pharm.D^a,b^, Daniel Bos, M.D, Ph.D^a,c^, Bruno H Stricker, M.D, Ph.D^a^, Fariba Ahmadizar, Pharm.D, Ph.D ^a,d^.

**Affiliations:**

1. Department of Epidemiology, Erasmus University Medical Center, Rotterdam, The Netherlands
2. Department of Internal Medicine, Division of Vascular Medicine and Pharmacology, Erasmus University Medical Center, Rotterdam, The Netherlands
3. Department of Radiology & Nuclear Medicine, Erasmus University Medical Center, Rotterdam, The Netherlands
4. Department of Data Science and Biostatistics, Julius global health, University Medical Center Utrecht, Utrecht, the Netherlands

**Corresponding author**:

Fariba Ahmadizar, Pharm.D, Ph.D

Department of Epidemiology, Erasmus University Medical Center, Rotterdam, The Netherlands

Postbus 2040| 3000 CA Rotterdam

Email: [f.ahmadizar@umcutrecht.nl](mailto:f.ahmadizar@umcutrecht.nl)

**Overview:**

1. **The search strategy**

- Table S1: Number of records that retrieved by the search strategy

1. **The inclusion and exclusion criteria**
2. **The PRISMA flow diagram**

- Figure S1: The PRISMA flow diagram

1. **Quality and risk of bias assessment**

- Table S2: The Newcastle-Ottawa quality assessment scale (NOS) for cohort studies.
- Table S3: The Newcastle-Ottawa quality assessment scale (NOS) for cross-sectional studies.
- Table S4: Cochrane Collaboration’s tool for quality and risk of bias assessment of randomized controlled trials (RCTs).

1. **PRISMA reporting checklist for systematic reviews**

- Table S5 PRISMA reporting checklist for systematic reviews

1. **Forest plots**

- Figures S2- S7

1. **Diagnostic statistics: influential cases analysis**

- Figure S8- S13

1. **Sub-group analyses**

- Table S6: sub-group analysis based on the defined daily dose of the control arm
- Table S7: sub-group analysis based on the industrial source of founding
- Table S8: sub-group analysis based on quality assessment results
- Table S9: Subgroup analyses of randomized controlled trials (RCT) based on follow-up time as longer/shorter than one year.

1. **Funnel plot, Egger’s regression test**

- Figure S14-S20
- Table S10: Egger’s regression test

1. **Synthesis without meta-analysis (SWiM)**

- Table S11: SWiM of cohort studies and the summary of effect estimates.
- Table S12: SWiM of cross-sectional studies and the summary of effect estimates.

1. **Grading of Recommendations Assessment, Development and Evaluation (GRADE) for the main outcomes of synthesis with and without meta-analysis**

- Table S13

1. **List of a number of potentially relevant studies not included in the systematic review, along with the reasons for exclusion.**

- Table S14

1. **References**
2. **The search strategy:**

Although a comprehensive approach consist of different cardiovascular medications, arterial locations, and imaging modalities was intended, focusing on the most frequent studies in terms of medication, artery location and imaging modality were designated (which are statins, coronary artery and CT scans respectively) to choose the optimal approach for the most clinically prevalent question being addressed.

**Embase.com**

('blood vessel calcification'/exp OR 'coronary artery calcium score'/exp OR ((calcium/de OR calcification/de) AND ('blood vessel'/exp OR 'coronary artery disease'/de)) OR (((arter* OR vessel* OR vascular* OR coronar* NEAR/10 (calcif* OR macrocalcif* OR calcium))):ab,ti,kw) AND ('diagnostic imaging'/de OR 'computer assisted tomography'/exp OR ((diagnos* NEAR/3 imag*) OR ((compute*) NEAR/3 tomogra*)):ab,ti,kw OR (ct OR mdct):ti) AND ('antilipemic agent'/de OR 'hypocholesterolemic agent'/de OR 'hydroxymethylglutaryl coenzyme A reductase inhibitor'/exp/de OR (((hypocholesterolemic* OR hypo-cholesterolemic* OR hypocholesterolaemic* OR hypo-cholesterolaemic* OR antilipem* OR anti-lipem* OR antilipaem* OR anti-lipaem* OR lipid-lower* OR cholesterol*) NEAR/3 (agent* OR drug* OR medication* OR therap* OR oral OR administrat* OR serum OR blood OR plasma)) OR ((hydroxymethylglutaryl OR hydroxyl-methylglutaryl OR hydroxyl-methyl-glutaryl OR hmg) NEAR/3 (coenzyme-A OR coa) NEAR/3 inhibitor*) OR statin* OR atorvastatin* OR bervastatin* OR cerivastatin* OR compactin* OR crilvastatin* OR dalvastatin* OR fluvastatin* OR fluindostatin* OR glenvastatin* OR lovastatin* OR mevinolin* OR monacolin-J* OR monacolin-L* OR pitavastatin* OR pravastatin* OR risedronic-acid* OR rosuvastatin* OR simvastatin* OR tenivastatin*):ab,ti,kw) NOT [conference abstract]/lim NOT ([animals]/lim NOT [humans]/lim) NOT ('case report'/de OR (case-report*):ti)

**Medline ALL Ovid**

(Vascular Calcification/ OR ((Calcium/ OR Calcification, Physiologic/ OR Calcinosis/) AND (exp Blood Vessels/ OR Coronary Artery Disease/)) OR (((arter* OR vessel* OR vascular* OR coronar*) ADJ10 (calcif* OR macrocalcif* OR calcium))).ab,ti,kf.) AND (Diagnostic Imaging/ OR exp Tomography, X-Ray Computed/ OR ((diagnos* ADJ3 imag*) OR ((compute*) ADJ3 tomogra*)).ab,ti,kf. OR (ct OR mdct).ti.) AND (Hypolipidemic Agents / OR exp Hydroxymethylglutaryl-CoA Reductase Inhibitors / OR (((hypocholesterolemic* OR hypo-cholesterolemic* OR hypocholesterolaemic* OR hypo-cholesterolaemic* OR antilipem* OR anti-lipem* OR antilipaem* OR anti-lipaem* OR lipid-lower* OR cholesterol*) ADJ3 (agent* OR drug* OR medication* OR therap* OR oral OR administrat* OR serum OR blood OR plasma)) OR ((hydroxymethylglutaryl OR hydroxyl-methylglutaryl OR hydroxyl-methyl-glutaryl OR hmg) ADJ3 (coenzyme-A OR coa) ADJ3 inhibitor*) OR statin* OR atorvastatin* OR bervastatin* OR cerivastatin* OR compactin* OR crilvastatin* OR dalvastatin* OR fluvastatin* OR fluindostatin* OR glenvastatin* OR lovastatin* OR mevinolin* OR monacolin-J* OR monacolin-L* OR pitavastatin* OR pravastatin* OR risedronic-acid* OR rosuvastatin* OR simvastatin* OR tenivastatin*).ab,ti,kf.) NOT (exp animals/ NOT humans/) NOT (case reports/ OR (case-report*).ti.)

**Web of Science SCI-EXPANDED & SSCI**

TS=(((((arter* OR vessel* OR vascular* OR coronar*) NEAR/10 (calcif* OR macrocalcif* OR calcium)))) AND (((diagnos* NEAR/2 imag*) OR ((compute* ) NEAR/2 tomogra*)) OR (ct OR mdct):ti) AND ((((hypocholesterolemic* OR hypo-cholesterolemic* OR hypocholesterolaemic* OR hypo-cholesterolaemic* OR antilipem* OR anti-lipem* OR antilipaem* OR anti-lipaem* OR lipid-lower* OR cholesterol*) NEAR/2 (agent* OR drug* OR medication* OR therap* OR oral OR administrat* OR serum OR blood OR plasma)) OR ((hydroxymethylglutaryl OR hydroxyl-methylglutaryl OR hydroxyl-methyl-glutaryl OR hmg) NEAR/2 (coenzyme-A OR coa) NEAR/2 inhibitor*) OR statin* OR atorvastatin* OR bervastatin* OR cerivastatin* OR compactin* OR crilvastatin* OR dalvastatin* OR fluvastatin* OR fluindostatin* OR glenvastatin* OR lovastatin* OR mevinolin* OR monacolin-J* OR monacolin-L* OR pitavastatin* OR pravastatin* OR risedronic-acid* OR rosuvastatin* OR simvastatin* OR tenivastatin*))) AND DT=( Article OR Review OR Letter OR Early Access) AND LA=(english)

**Cochrane CENTRAL Register of Trials**

((((arter* OR vessel* OR vascular* OR coronar*) NEAR/10 (calcif* OR macrocalcif* OR calcium))):ab,ti) AND (((diagnos* NEAR/3 imag*) OR ((compute*) NEAR/3 tomogra*)):ab,ti OR (ct OR mdct):ti) AND ((((hypocholesterolemic* OR hypo NEXT cholesterolemic* OR hypocholesterolaemic* OR hypo NEXT cholesterolaemic* OR antilipem* OR anti NEXT lipem* OR antilipaem* OR anti NEXT lipaem* OR lipid NEXT lower* OR cholesterol*) NEAR/3 (agent* OR drug* OR medication* OR therap* OR oral OR administrat* OR serum OR blood OR plasma)) OR ((hydroxymethylglutaryl OR hydroxyl NEXT methylglutaryl OR hydroxyl NEXT methyl NEXT glutaryl OR hmg) NEAR/3 (coenzyme NEXT A OR coa) NEAR/3 inhibitor*) OR statin* OR atorvastatin* OR bervastatin* OR cerivastatin* OR compactin* OR crilvastatin* OR dalvastatin* OR fluvastatin* OR fluindostatin* OR glenvastatin* OR lovastatin* OR mevinolin* OR monacolin NEXT J* OR monacolin NEXT L* OR pitavastatin* OR pravastatin* OR risedronic NEXT acid* OR rosuvastatin* OR simvastatin* OR tenivastatin*):ab,ti) NOT "conference abstract":pt

Table S1: Number of records retrieved by the search strategy

| **Database searched** | **via** | **Years of coverage** | **Records** | **Records after duplicates removed** |
| --- | --- | --- | --- | --- |
| Embase | Embase.com | 1971 - Present | 1286 | 1269 |
| Medline ALL | Ovid | 1946 - Present | 460 | 55 |
| Web of Science Core Collection* | Web of Knowledge | 1975 - Present | 568 | 180 |
| Cochrane Central Register of Controlled Trials | Wiley | 1992 - Present | 63 | 9 |
| **Total** | | | **2377** | **1513** |

*Science Citation Index Expanded (1975-present) ; Social Sciences Citation Index (1975-present) ; Arts & Humanities Citation Index (1975-present) ; Conference Proceedings Citation Index- Science (1990-present) ; Conference Proceedings Citation Index- Social Science & Humanities (1990-present) ; Emerging Sources Citation Index (2015-present)

1. **Inclusion and exclusion criteria:**

We included observational research, including cross-sectional and longitudinal studies and randomized controlled trials (RCTs), that studied the association between statin use and coronary artery calcification (CAC).

Other criteria for inclusion included:

1. Studied human adult subjects.
2. Statins-group or HMG CoA reductase inhibitors, ATC code: C10AA, include at least one of the following medications: Atorvastatin, fluvastatin, lovastatin, pitavastatin, pravastatin, rosuvastatin, and simvastatin
3. A computed tomography (CT) scan detected coronary arterial calcification (CAC) score. CAC score might be dichotomous or continuous in format.  Continuous-format scoring could be in any unit of measurement, such as volumetric (mm3) or Agatston score.
4. Observational studies that reported an effect size of the association between statins and CAC score.
5. RCTs measured CAC score before and after the statin as an intervention.
6. RCTs with at least an active interventional arm consisting solely of statins and a control arm consisting of one of the following:
   1. Placebo or no treatment arm
   2. Different defined daily dose of statins

We excluded if:

1. Statins were examined in combination with other medicines, not on their own.
2. Calcification was examined in non-coronary arterial regions rather than coronary.
3. CAC was detected using imaging modalities other than a CT scan, such as MRI or intravascular ultrasound (IVUS).
4. Descriptive observational studies which have not reported any effect size for the association.
5. Post-hoc analysis of the same data that was previously published and included.
6. Non-randomized clinical trials.
7. Other study designs rather than observational and RCTs and other publication types such as case series, case reports, brief reports, letters, reviews, meta-analyses, and theoretical, biological, and experimental studies.
   - - 1. **PRISMA flow diagram**

Figure S1: PRISMA flow diagram

**Identification of studies via other methods**

**Identification of studies via databases and registers**

Records identified from

Databases and

Registers (n =2377)

Records identified through manual search, gray literature, and reference of the references (n-=83).

Duplicate records removed

(n = 864).

**Identification**

Records excluded by title and abstract manual screening

(n = 1334).

Records screened

(n = 1513).

Records sought for retrieval (n =45).

Reports not retrieved (n=30):

Conference abstract (n=9),

Full text not accessible (n=1)

Letters, commentary,

brief report (n=6).

Other imaging modalities (n=14).

Reports sought for retrieval (n =179).

**Screening**

Reports assessed for eligibility (n =2).

Reports assessed for eligibility (n =149).

Reports excluded (n=108):

Descriptive studies (n =21),

Statin in combination (n=2),

No comparator group in trial (n=5),

Post hoc studies (n=2),

Heart Valve=(6),

Other reasons or reasons for exclusion involved at least 2 of the above (n=72).

Studies included in review (n =41).

Studies included in meta-analysis (n=32).

**Included**

- - - 1. **Quality and risk of bias assessment**

Quality assessments were carried out by two investigators (M.N.S and S.M.J) independently. The Newcastle-Ottawa quality assessment scale (NOS) (1, 2) was applied to assess the quality of observational studies. The quality of each RCT was assessed using the Cochrane risk of bias tool (3).

Table S2: The Newcastle-Ottawa quality assessment scale (NOS) for longitudinal studies.

| Cohort Study | | Selection | | | | Comparability | Outcome | | |  |
| --- | --- | --- | --- | --- | --- | --- | --- | --- | --- | --- |
|  |  | Representative of the exposed cohort | Selection of the non-exposed cohort | Ascertainment of the exposure | Demonstration that the outcome of interest was not present at the start of the study | Comparability of the cohort on the basis of the design or analysis | Assessment of outcome | Was follow-up long enough for outcome of interest | Adequacy of follow-up of cohorts |  |
|  | Shemesh J, 2004 | 1 | 1 | 1 | 1 | 1 | 1 | 1 | 1 | 8/9 |
|  | Hsia J, 2004 | 1 | 1 | 0 | 1 | 1 | 1 | 1 | 1 | 7/9 |
|  | Budoff MJ, 2005 | 1 | 1 | 1 | 1 | 0 | 1 | 1 | 1 | 7/9 |
|  | Raggi P, 2005 | 1 | 1 | 0 | 1 | 1 | 1 | 1 | 1 | 7/9 |
|  | Anand DV, 2007 | 1 | 1 | 0 | 1 | 1 | 1 | 1 | 1 | 7/9 |
|  | Elkeles RS, 2008 | 1 | 1 | 2 | 1 | 1 | 1 | 1 | 1 | 9/9 |
|  | Hoffmann H, 2010 | 0 | 1 | 1 | 1 | 1 | 1 | 1 | 1 | 7/9 |
|  | Marchal C, 2012 | 1 | 1 | 1 | 1 | 1 | 1 | 1 | 1 | 8/9 |
|  | Zeb I, 2013 | 1 | 1 | 0 | 1 | 1 | 1 | 1 | 1 | 7/9 |
|  | Chen Z, 2017 | 1 | 1 | 0 | 1 | 1 | 1 | 1 | 1 | 7/9 |
|  | Smit JM, 2020 | 1 | 1 | 1 | 1 | 1 | 1 | 1 | 1 | 8/9 |
|  | Karpouzas GA, 2020 | 1 | 1 | 2 | 1 | 1 | 1 | 1 | 1 | 9/9 |
|  | Pechlivanis et al , 2021 | 1 | 1 | 1 | 1 | 1 | 1 | 1 | 1 | 8/9 |

Table S3: The Newcastle-Ottawa quality assessment scale (NOS) for cross-sectional studies (adapted version)

| Cross-sectional Study | | Selection | | | | Comparability | Outcome | | Total |
| --- | --- | --- | --- | --- | --- | --- | --- | --- | --- |
|  |  | Representativeness of the sample | Sample size | Non-respondents | Ascertainment of the exposure | The subjects in different outcome groups are comparable | Assessment of the outcome | Statistical test |  |
|  | Elkeles RS, 2004 | 1 | 1 | 1 | 1 | 1 | 2 | 1 | 8/9 |
|  | NguyenPTH, 2007 | 1 | 1 | 1 | 1 | 1 | 2 | 1 | 8/9 |
|  | Hosseinsabet, 2009 | 1 | 1 | 0 | 1 | 1 | 2 | 1 | 7/9 |
|  | Cheng VY, 2010 | 1 | 1 | 0 | 1 | 1 | 2 | 1 | 7/9 |
|  | Jung CH, 2010 | 0 | 0 | 0 | 1 | 1 | 2 | 1 | 5/9 |
|  | Jeon GH, 2010 | 1 | 1 | 1 | 1 | 1 | 2 | 1 | 8/9 |
|  | Hamer et al, 2012 | 1 | 1 | 1 | 0 | 1 | 2 | 1 | 7/9 |
|  | Nakazato R, 2012 | 1 | 1 | 1 | 1 | 1 | 2 | 1 | 8/9 |
|  | Greif M, 2013 | 1 | 1 | 1 | 1 | 1 | 1 | 1 | 7/9 |
|  | Rodriguez K, 2015 | 1 | 1 | 1 | 1 | 1 | 2 | 1 | 7/9 |
|  | Shikada T, 2015 | 1 | 1 | 0 | 1 | 1 | 2 | 1 | 7/9 |
|  | Panh L, 2017 | 1 | 1 | 0 | 1 | 1 | 2 | 1 | 7/9 |
|  | Lee D, 2018 | 1 | 1 | 1 | 2 | 1 | 2 | 1 | 9/9 |
|  | Drouin-Chartier JP,2020 | 1 | 1 | 1 | 2 | 1 | 2 | 1 | 9/9 |
|  | Zhelyazkova-Savova MD, 2021 | 0 | 1 | 0 | 1 | 1 | 1 | 1 | 5/9 |
|  | Beland-Bonenfant, 2021 | 1 | 0 | 1 | 1 | 1 | 1 | 1 | 6/9 |

Table S4: Study quality and risk of bias assessment using Cochrane collaboration’s tool for interventional studies.

| Trial | | Criteria | | | | | | | |
| --- | --- | --- | --- | --- | --- | --- | --- | --- | --- |
|  |  | Sequence generation | Allocation concealment | Blinding of participant or personnel | Blinding of outcome  assessment | Incomplete  outcome data | Selective outcome  reporting | Other potential threats | Overall risk of bias |
|  | Houslay et al, 2006 | L | U | L | L | H | L | L | L |
|  | Terry et al, 2007 | U | U | U | L | L | L | L | L |
|  | Dichtl et al, 2008 | U | U | H | H | L | L | L | H |
|  | Petri et al, 2011 | L | U | L | L | H | U | U | H |
|  | Plazak et al, 2011 | L | U | U | L | L | L | L | L |
|  | Lemos et al, 2013 | L | U | H | H | L | L | L | U |
|  | Lo et al, 2015 | L | U | L | L | L | L | L | L |
|  | Raggi et al, 2005 | U | U | L | L | L | L | L | L |
|  | Schmermund et al, 2006 | L | U | L | L | L | L | L | L |
|  | Auscher et al, 2015 | U | U | H | H | L | L | U | U |
|  | Yazbek et al, 2016 | L | L | H | H | L | L | L | L |
|  | Miyoshi et al, 2018 | L | U | H | H | U | L | U | U |

H: High risk of bias, L: Low risk of bias, U:Unclear risk of bias

- - - 1. **PRISMA reporting checklist for systematic reviews**

Table S5: PRISMA reporting checklist for systematic reviews

| **Section and Topic** | **Item #** | **Checklist item** | **Reported on Page:** |
| --- | --- | --- | --- |
| **TITLE** | | |  |
| Title | 1 | Identify the report as a systematic review. | 1 |
| **ABSTRACT** | | |  |
| Abstract | 2 | See the PRISMA 2020 for Abstracts checklist. | 2 |
| **INTRODUCTION** | | |  |
| Rationale | 3 | Describe the rationale for the review in the context of existing knowledge. | 4 |
| Objectives | 4 | Provide an explicit statement of the objective(s) or question(s) the review addresses. | 5 |
| **METHODS** | | |  |
| Eligibility criteria | 5 | Specify the inclusion and exclusion criteria for the review and how studies were grouped for the syntheses. | S* 5 |
| Information sources | 6 | Specify all databases, registers, websites, organisations, reference lists and other sources searched or consulted to identify studies. Specify the date when each source was last searched or consulted. | S 2-4 |
| Search strategy | 7 | Present the full search strategies for all databases, registers and websites, including any filters and limits used. | S 2-4 |
| Selection process | 8 | Specify the methods used to decide whether a study met the inclusion criteria of the review, including how many reviewers screened each record and each report retrieved, whether they worked independently, and if applicable, details of automation tools used in the process. | 5 and 6 |
| Data collection process | 9 | Specify the methods used to collect data from reports, including how many reviewers collected data from each report, whether they worked independently, any processes for obtaining or confirming data from study investigators, and if applicable, details of automation tools used in the process. | 5 and 6 |
| Data items | 10a | List and define all outcomes for which data were sought. Specify whether all results that were compatible with each outcome domain in each study were sought (e.g. for all measures, time points, analyses), and if not, the methods used to decide which results to collect. | Table1-3 |
|  | 10b | List and define all other variables for which data were sought (e.g. participant and intervention characteristics, funding sources). Describe any assumptions made about any missing or unclear information. | Table1-3 |
| Study risk of bias assessment | 11 | Specify the methods used to assess risk of bias in the included studies, including details of the tool(s) used, how many reviewers assessed each study and whether they worked independently, and if applicable, details of automation tools used in the process. | 5 |
| Effect measures | 12 | Specify for each outcome the effect measure(s) (e.g. risk ratio, mean difference) used in the synthesis or presentation of results. | 5, 6, and 7 |
| Synthesis methods | 13a | Describe the processes used to decide which studies were eligible for each synthesis (e.g. tabulating the study intervention characteristics and comparing against the planned groups for each synthesis (item #5)). | 6 and 7 |
|  | 13b | Describe any methods required to prepare the data for presentation or synthesis, such as handling of missing summary statistics, or data conversions. | 6 and 7 |
|  | 13c | Describe any methods used to tabulate or visually display results of individual studies and syntheses. | 6 and 7 |
|  | 13d | Describe any methods used to synthesize results and provide a rationale for the choice(s). If meta-analysis was performed, describe the model(s), method(s) to identify the presence and extent of statistical heterogeneity, and software package(s) used. | 6 and 7 |
|  | 13e | Describe any methods used to explore possible causes of heterogeneity among study results (e.g. subgroup analysis, meta-regression). | 6 and 7 |
|  | 13f | Describe any sensitivity analyses conducted to assess robustness of the synthesized results. | 6 and 7 |
| Reporting bias assessment | 14 | Describe any methods used to assess risk of bias due to missing results in a synthesis (arising from reporting biases). | 6 and 7 |
| Certainty assessment | 15 | Describe any methods used to assess certainty (or confidence) in the body of evidence for an outcome. | 6 and 7 |
| **RESULTS** | | |  |
| Study selection | 16a | Describe the results of the search and selection process, from the number of records identified in the search to the number of studies included in the review, ideally using a flow diagram. | 8 |
|  | 16b | Cite studies that might appear to meet the inclusion criteria, but which were excluded, and explain why they were excluded. | S-10 |
| Study characteristics | 17 | Cite each included study and present its characteristics. | 8 and Table 1-3 |
| Risk of bias in studies | 18 | Present assessments of risk of bias for each included study. | S-5 and S-6 |
| Results of individual studies | 19 | For all outcomes, present, for each study: (a) summary statistics for each group (where appropriate) and (b) an effect estimate and its precision (e.g. confidence/credible interval), ideally using structured tables or plots. | 8 and Figures2-8 |
| Results of syntheses | 20a | For each synthesis, briefly summarise the characteristics and risk of bias among contributing studies. | 8 |
|  | 20b | Present results of all statistical syntheses conducted. If meta-analysis was done, present for each the summary estimate and its precision (e.g. confidence/credible interval) and measures of statistical heterogeneity. If comparing groups, describe the direction of the effect. | 8 |
|  | 20c | Present results of all investigations of possible causes of heterogeneity among study results. | 9 |
|  | 20d | Present results of all sensitivity analyses conducted to assess the robustness of the synthesized results. | 9 and S-11 to S-23 |
| Reporting biases | 21 | Present assessments of risk of bias due to missing results (arising from reporting biases) for each synthesis assessed. | Table 4- GRADE |
| Certainty of evidence | 22 | Present assessments of certainty (or confidence) in the body of evidence for each outcome assessed. | Table 4- GRADE |
| **DISCUSSION** | | |  |
| Discussion | 23a | Provide a general interpretation of the results in the context of other evidence. | 10 |
|  | 23b | Discuss any limitations of the evidence included in the review. | 12-13 |
|  | 23c | Discuss any limitations of the review processes used. | 11 |
|  | 23d | Discuss implications of the results for practice, policy, and future research. | 12 |
| **OTHER INFORMATION** | | |  |
| Registration and protocol | 24a | Provide registration information for the review, including register name and registration number, or state that the review was not registered. | 5 |
|  | 24b | Indicate where the review protocol can be accessed, or state that a protocol was not prepared. | 5 |
|  | 24c | Describe and explain any amendments to information provided at registration or in the protocol. | S-search strategy |
| Support | 25 | Describe sources of financial or non-financial support for the review, and the role of the funders or sponsors in the review. | Non |
| Competing interests | 26 | Declare any competing interests of review authors. | Declared |
| Availability of data, code and other materials | 27 | Report which of the following are publicly available and where they can be found: template data collection forms; data extracted from included studies; data used for all analyses; analytic code; any other materials used in the review. | 14 |

- - - 1. **Forrest Plots**

Figure S2: Forrest plot for cohort studies which with binary effect metric.


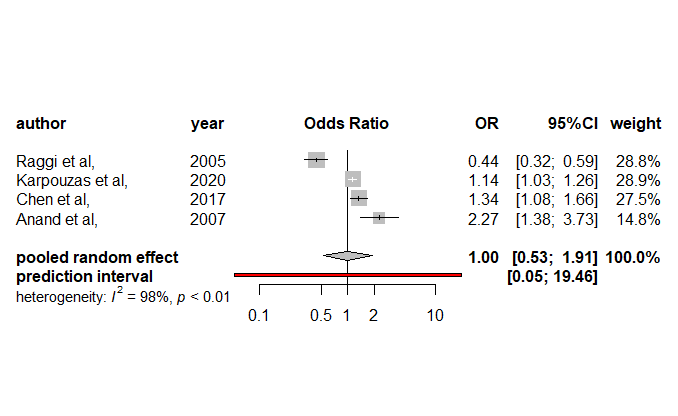
The pooled random effect is the odds ratio (OR) and 95% confidence interval of –having- progression in coronary artery calcification for statin use.

CI: confidence interval.

Figure S3: Forrest plot for cohort studies with continuous effect metrics.


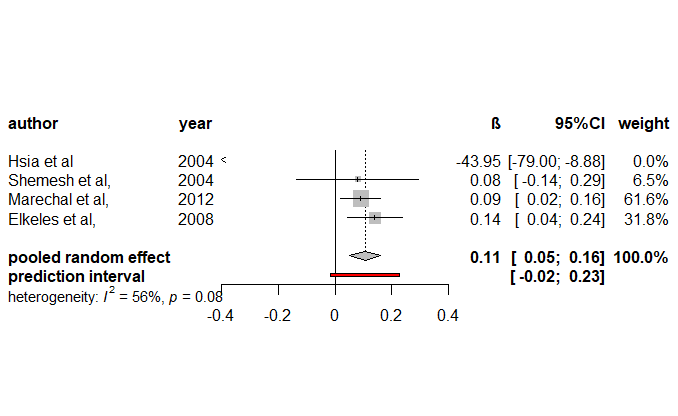


The pooled random effect is ẞ coefficients and 95% confidence interval of the coronary artery calcification quantified as Agatston score for statins. The outcomes of associations in the studies by Shemesh et al., Marechal et al., and Elkeles et al. were log-transformed before they ran the analysis.

CI: confidence interval.

Figure S4: Forrest plot for cross-sectional studies with binary effect metric.


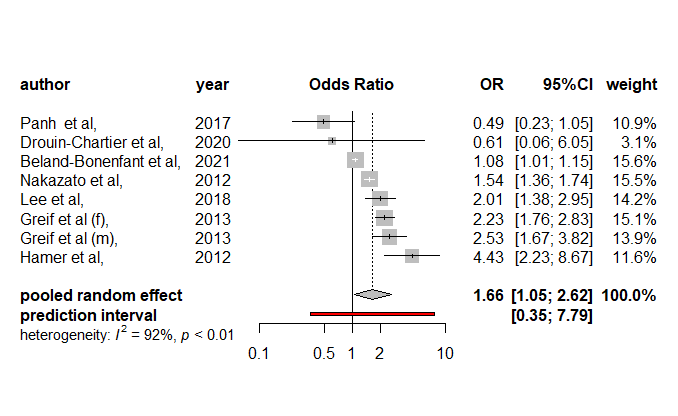


The pooled random effect is the odds ratio (OR) and 95%confident interval of the presence of coronary artery calcification for using statins.

CI: confidence interval.

Figure S5: Forrest plot for cross-sectional studies with continuous effect metrics.


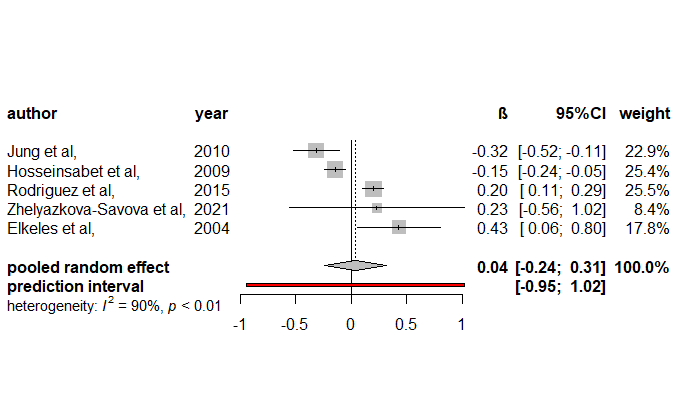


The pooled random effect is ẞ coefficients and 95% confidence interval of the coronary artery calcification score quantified as Agatston score for statin use. The outcomes of associations in the studies by Jung et al., Hosseinsabet et al., Rodrigez et al. and Elkeles et al. were log-transformed before they ran the analysis.

CI: confidence interval.

Figure S6: Forrest plot for randomized controlled trials (RCTs) quantified coronary artery calcification as Agatston score.


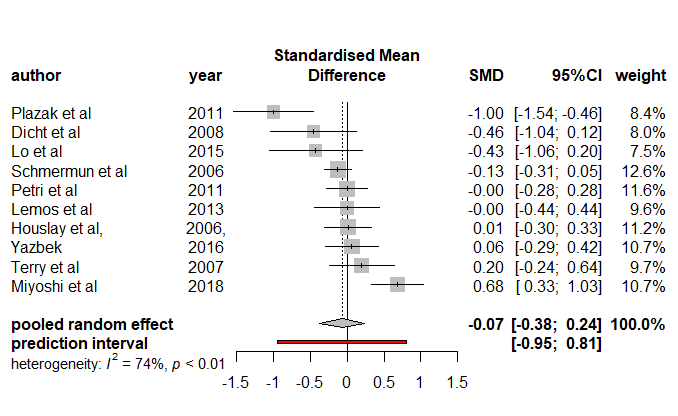


The pooled random effect is a standardized mean difference (SMD) and 95% confidence interval for the effect of statins on coronary artery calcification quantified as the Agatston score.

CI: confidence interval.

Figure S7: Forrest plot for randomized controlled trials (RCTs) quantified coronary artery calcification as volume.


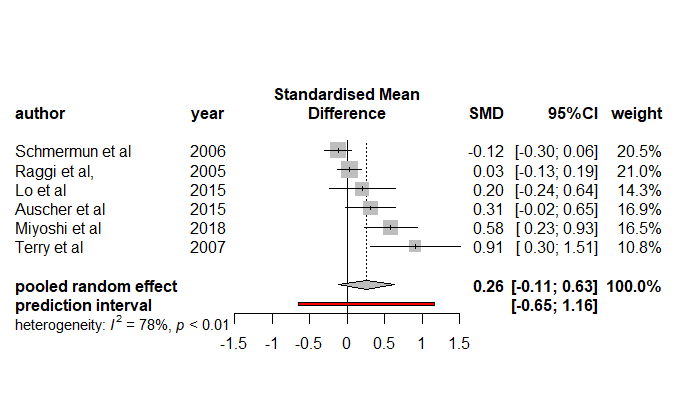
The pooled random effect is a standardized mean difference (SMD), and 95% confidence interval for the effect of statins on coronary artery calcification quantified as volume.

CI: confidence interval.

- - - 1. **Influential cases analyses (sensitivity analyses):**

Figure S8: Leave one out analysis of cohort studies with binary outcome metric (OR) A: sorted by I^2^, B: sorted by effect size.

A:
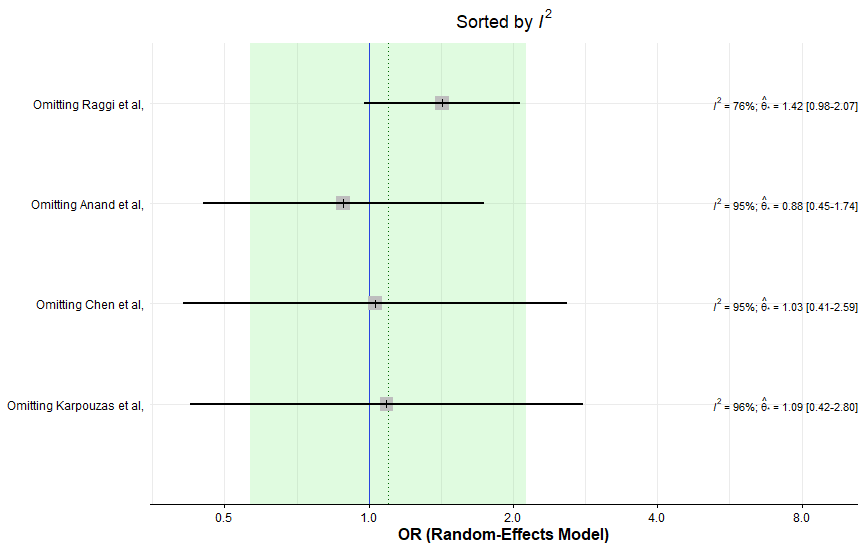


B:
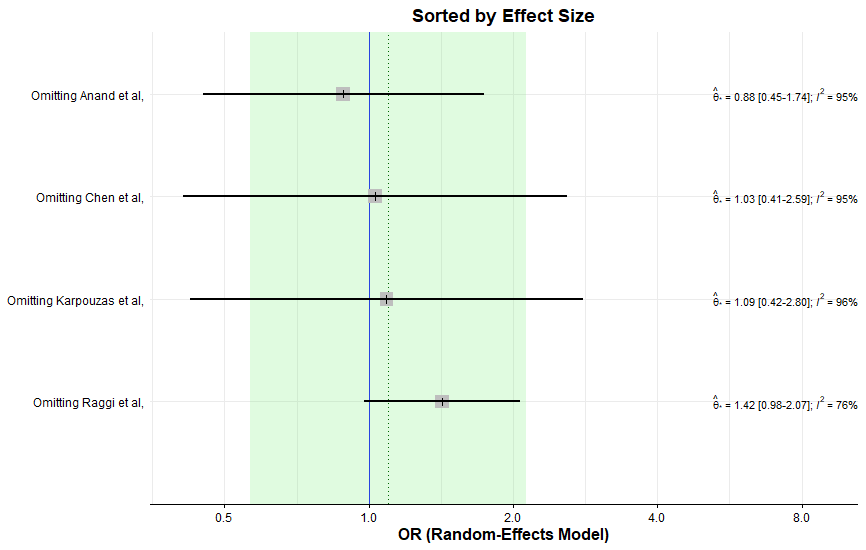


Figure S9: Leave one out analysis of cohort studies with continuous outcome metric (ẞ), A: sorted by I^2^ , B: sorted by effect size.

A:
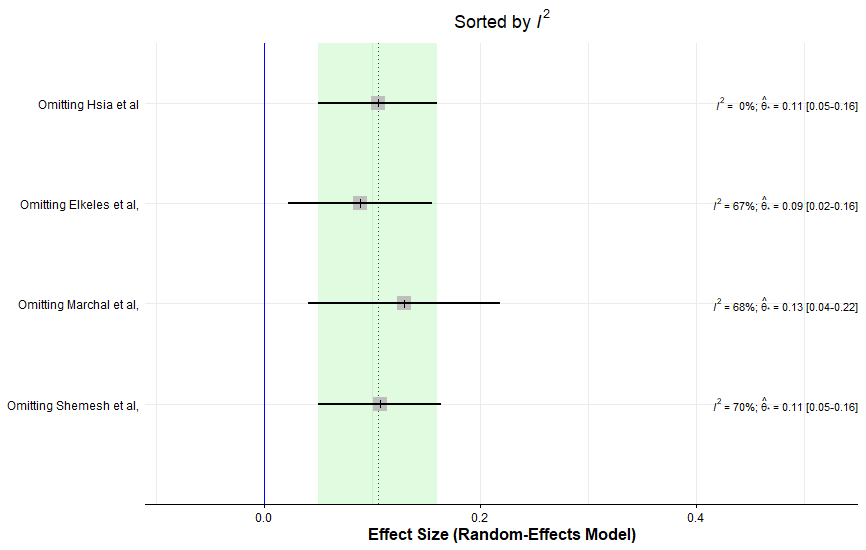


B:
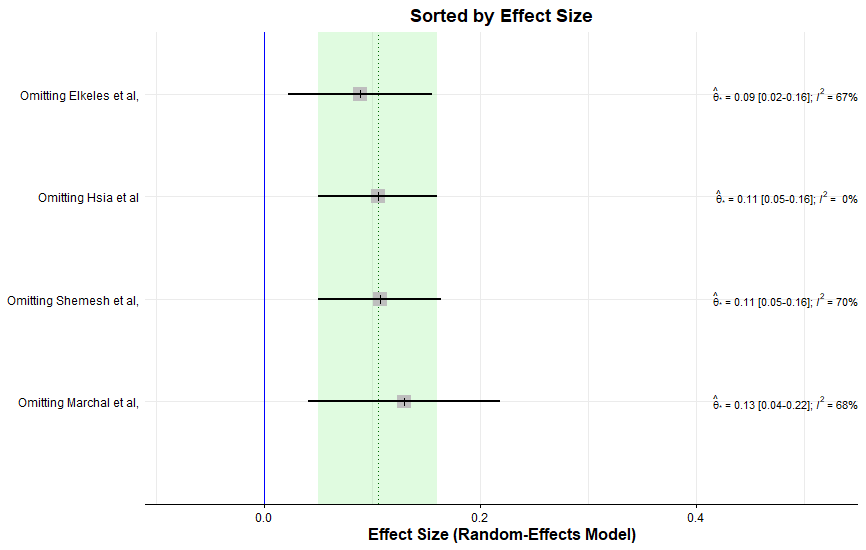


Figure S10: Leave one out analysis of cross-sectional studies with binary outcome metric (OR), A: sorted by I^2^ , B: sorted by effect size.

A:
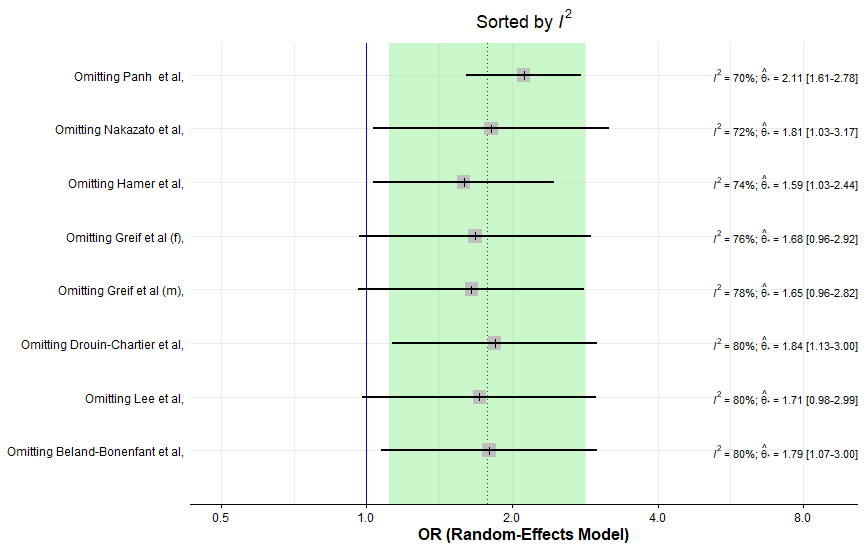


B**:** **
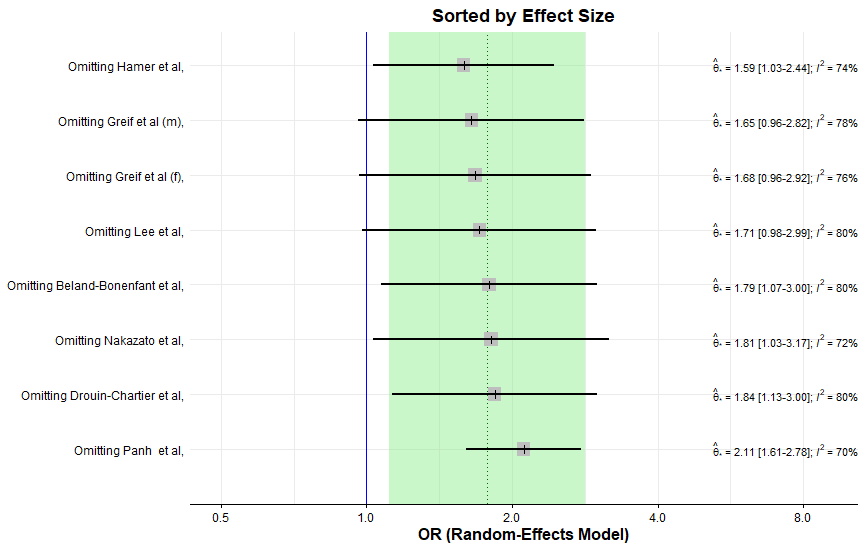
**

Figure S11: Leave one out analysis of cross sectional studies with continuous outcome metric (ẞ), A: sorted by I^2^ , B: sorted by effect size.

A: **
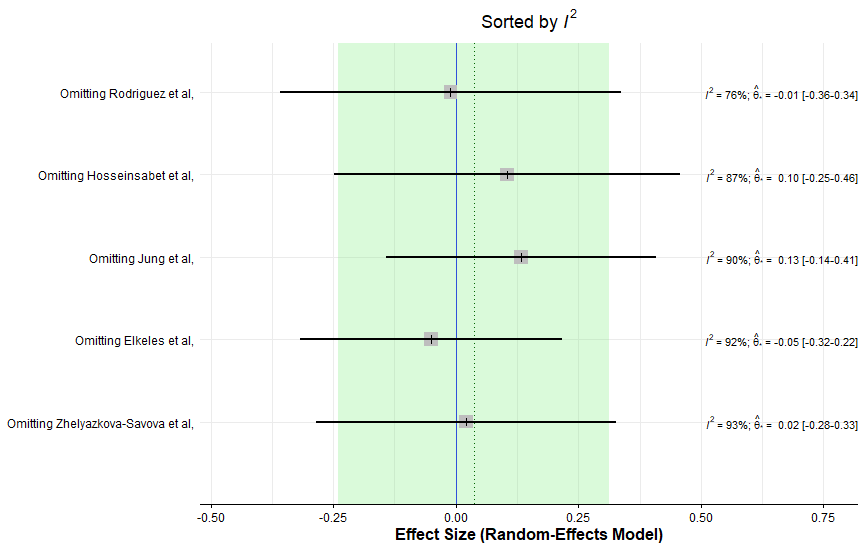
**

**
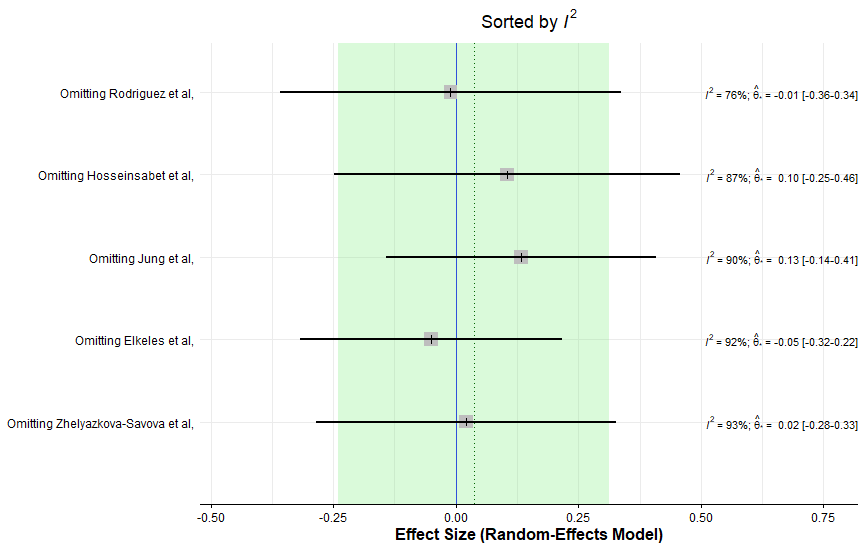
**

B:

Figure S12: Leave one out analysis of randomized control trials qualified coronary artery calcification as Agatston score, A: sorted by I^2^ ,B: sorted by effect size.

A:
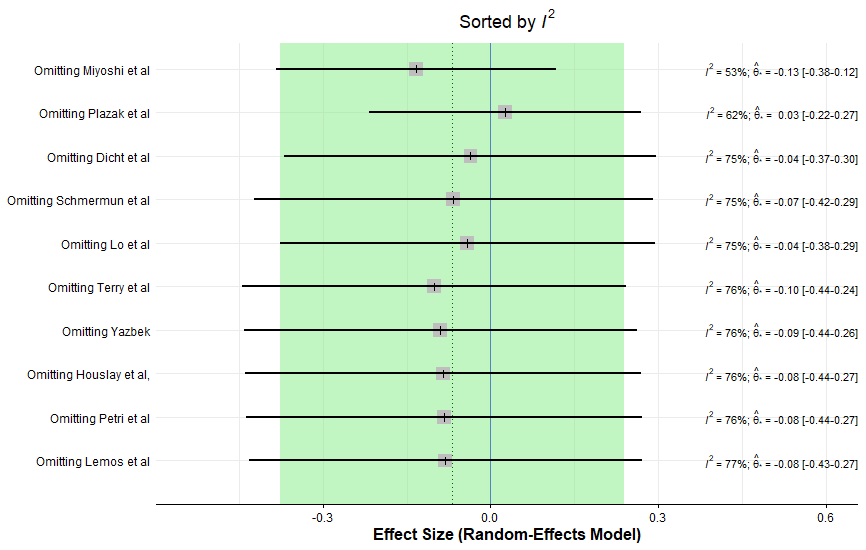


B:
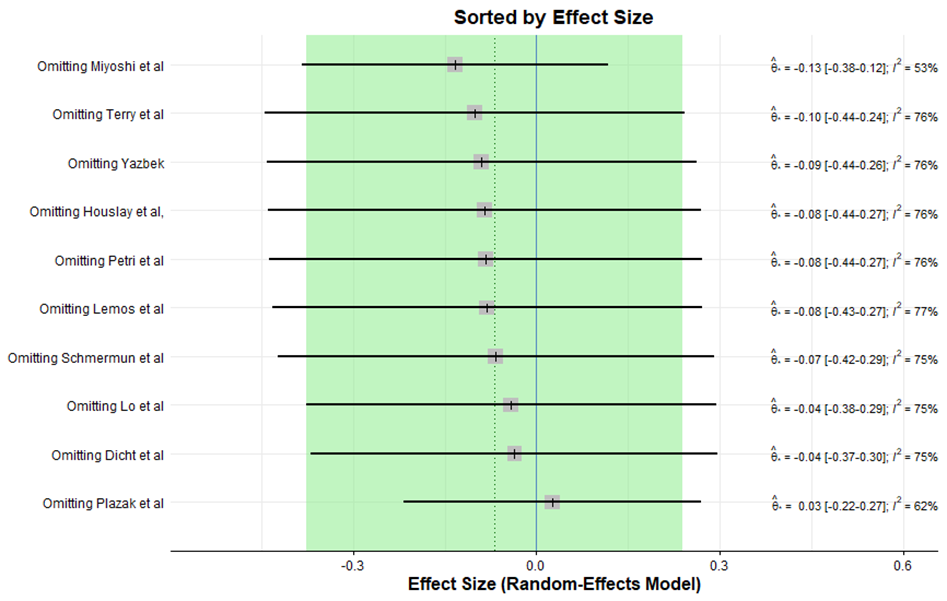


Figure S13 : Leave one out analysis of randomized control trials which qualified coronary artery calcification as volume, A: sorted by I^2^ , B: sorted by effect size.

A: **
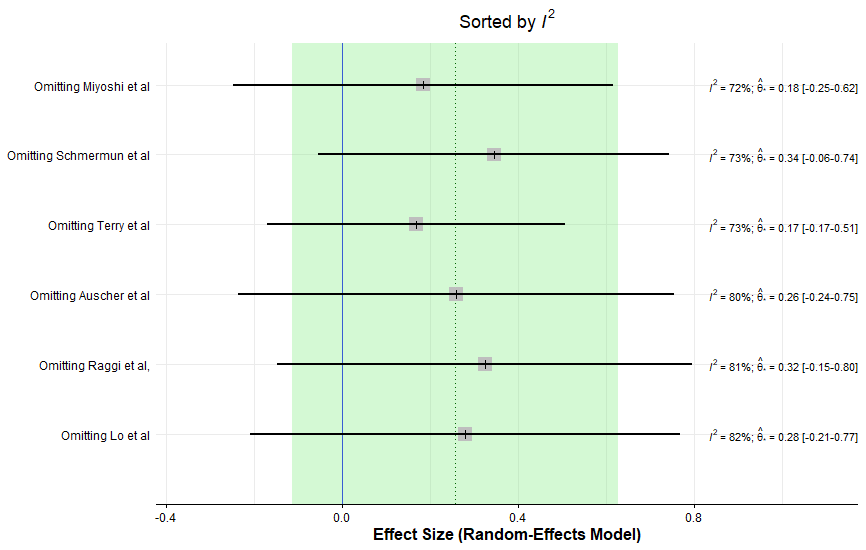
**

B:
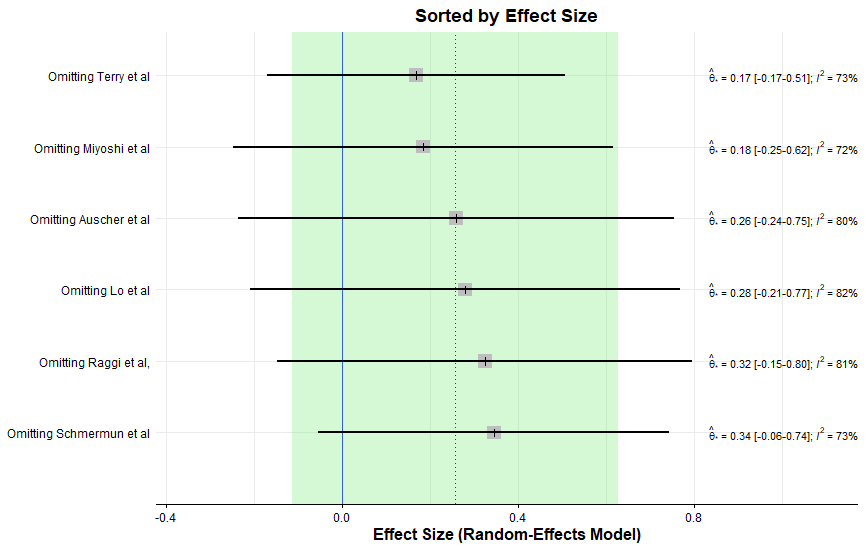


Table S6: Subgroup analyses of randomized controlled trials (RCT) based on the defined daily dose of the control arm as either placebo/no-treatment or low-dose statin.

| Model,  Control arm, | | Number of RCTs | Pooled effect size | 95%CI of the effect,  95%PI of the effect | I^2^ | 95%CI* |
| --- | --- | --- | --- | --- | --- | --- |
| 1 | Meta-analysis of RCTs,  Control arm: Placebo or no treatment | 8 | -0.02 | [-0.47; 0.42],  [-1.25; 1.20] | 79.6% | [-0.83 to 0.21]** |
|  | Outliers and influential cases removed^1^ | 7 | -0.14 | [-0.50; 0.22],  [-0.95; 0.67] | 59.8% |  |
| 2 | Meta-analysis of RCTs,  Control arm: Low dose statin | 4 | 0.17 | [-0.32; 0.66],  [-1.17; 1.51] | 77.5% |  |

CAC: coronary artery calcification, SMD: standardized mean difference, CI: confidence interval.

1: Removed as an outlier: "Terry et al"

*The unpaired t-test after having outliers and influential cases removed, if detected any.

**: The two-tailed P value equals 0.21 and the difference is not statistically significant.

Table S7: Subgroup analyses of randomized controlled trials (RCT) based on their industrial source of founding

| Model,  Industrial source of founding | | Number of included RCTs | Pooled effect size | 95%CI of the effect,  95%PI of the effect | I^2^ | 95%CI * |
| --- | --- | --- | --- | --- | --- | --- |
| 1 | Meta-analysis of RCTs,  with industrial source of founding | 5 | 0.25 | [-0.29; 0.78],  [-1.12; 1.62] | 84.4% | [-0.21 to 0.91]* |
| 2 | Meta-analysis of RCTs,  without industrial source of founding | 7 | -0.10 | [-0.50; 0.30], [-1.09; 0.90] | 69.1% |  |

CAC: coronary artery calcification, SMD: standardized mean difference, CI: confidence interval.

* The two-tailed P value of the unpaired t-test, with no detected outliers and influential cases, equals 0.19 and the difference is not statistically significant.

Table S8: Subgroup analyses of randomized controlled trials (RCT) based on their quality assessment results (QAR) as low/high/unclear risk of bias.

| Model,  QAR, | | Number of included RCTs | Pooled SMD | 95%CI of the effect,  95%PI of the effect | I^2^ | P-value* |
| --- | --- | --- | --- | --- | --- | --- |
| 1 | Meta-analysis of RCTs  QAR: low risk of bias | 7 | 0.02 | [-0.48; 0.51],  [-1.29; 1.32] | 80.4% | 0.17* |
| 2 | Meta-analysis of RCTs  QAR: high risk of bias | 2 | -0.16 | [-2.9; 2.60],  [n/a] | 48.8% |  |
| 3 | Meta-analysis of RCTs  QAR: unclear | 3 | 0.32 | [-0.38; 1.02], [-2.88; 3.52] | 51.5% |  |

CAC: coronary artery calcification, SMD: standardized mean difference, CI: confidence interval, n/a: not available; due to few numbers of included studies.

* The two-tailed P value of the ANOVA test, with no detected outliers and influential cases, is not statistically significant.

Table S9: Subgroup analyses of randomized controlled trials (RCT) based on follow-up time as longer/shorter than one year.

| Model, | | Number of included RCTs | Pooled effect size | 95%CI of the effect,  95%PI of the effect | I^2^ | 95%CI * |
| --- | --- | --- | --- | --- | --- | --- |
| 1 | Meta-analysis of RCTs,  with longer than one year of follow up | 4 | -0.041 | [-0.29; 0.21] | 0.0% | [-0.73 to 0.31]** |
| 2 | Meta-analysis of RCTs,  with 1 year or shorter follow up | 8 | 0.128 | [-0.32; 0.58],  [-1.13; 1.39] | 83.7% |  |
|  | Outliers and influential cases removed^1^ | 7 | 0.25 | [-0.08; 0.58],  [-0.61, 1.11] | 78.1% |  |

CAC: coronary artery calcification, SMD: standardized mean difference, CI: confidence interval. * Unpaired t-test after having outliers and influential cases removed.

**The two-tailed P value equals 0.38 which is not statistically significant.

1: Removed as outlier: "Plazak et al"

- - - 1. **Funnel plots**

Effect estimates from individual studies against the measure of size and precision (standard error) of each effect. Plots have been developed for each met-analysis model. Contour-enhanced funnel plot displayed for the models with significant small-study effect.

Figure S14:

**
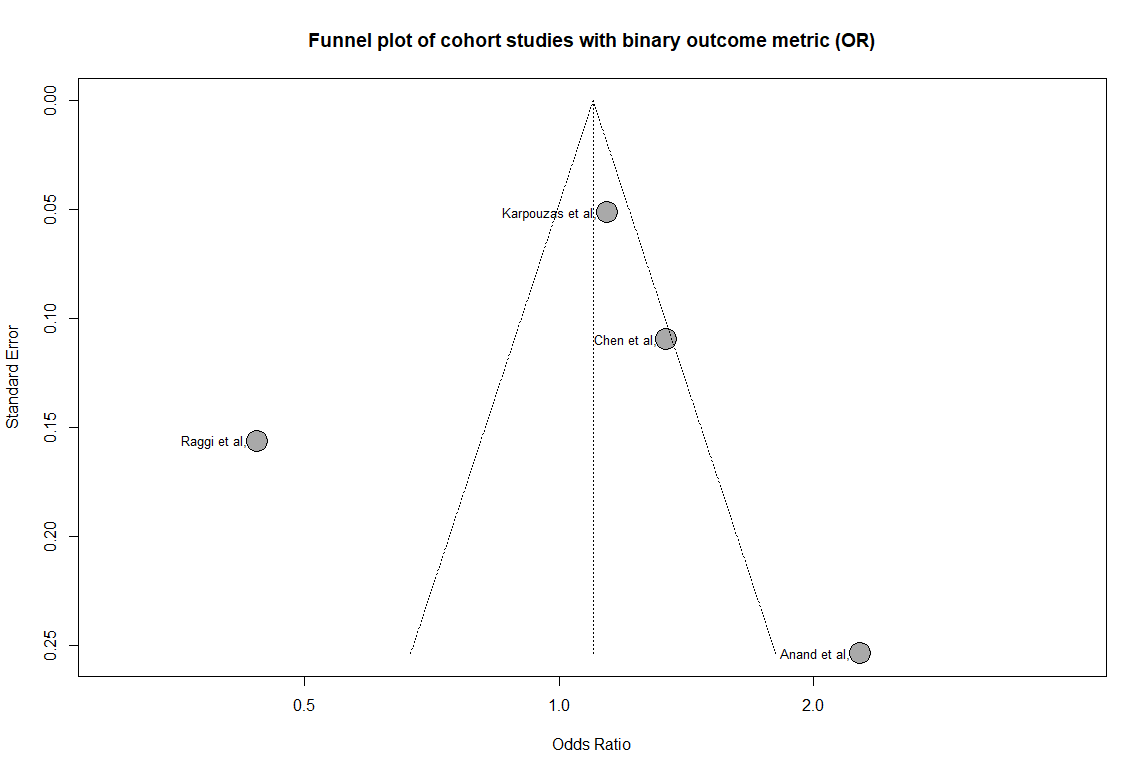
**

Figure S15:

**
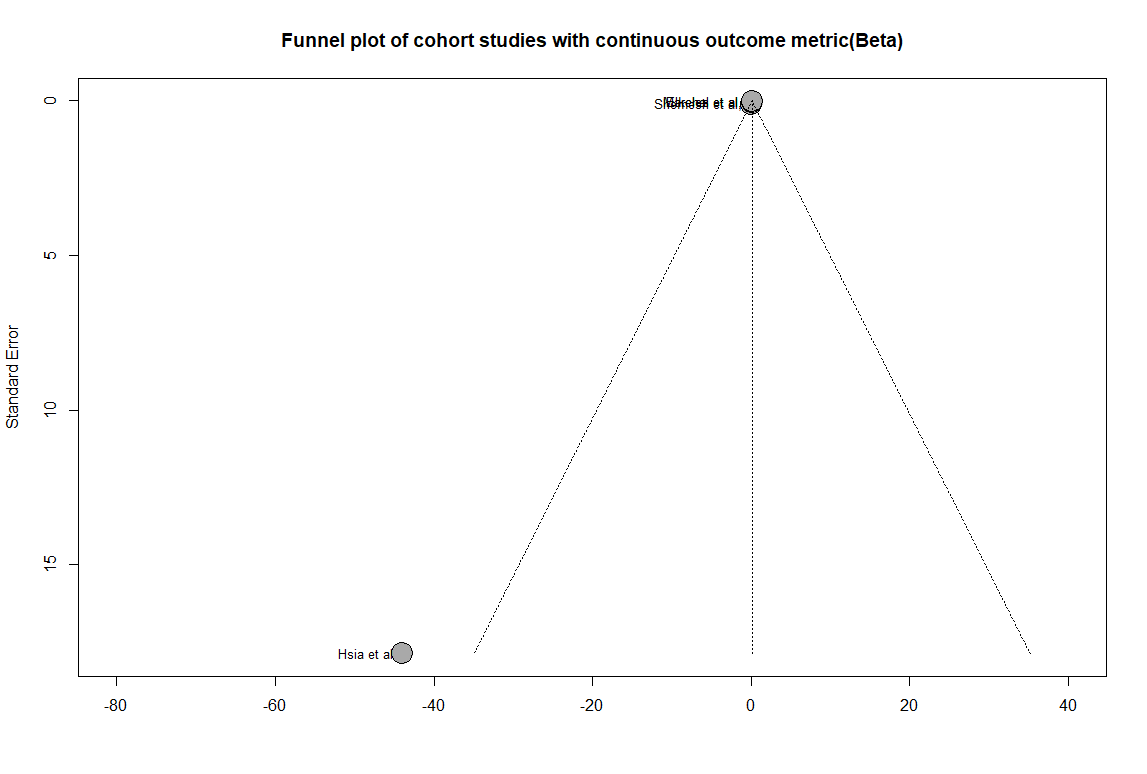
**

Figure S16:

**
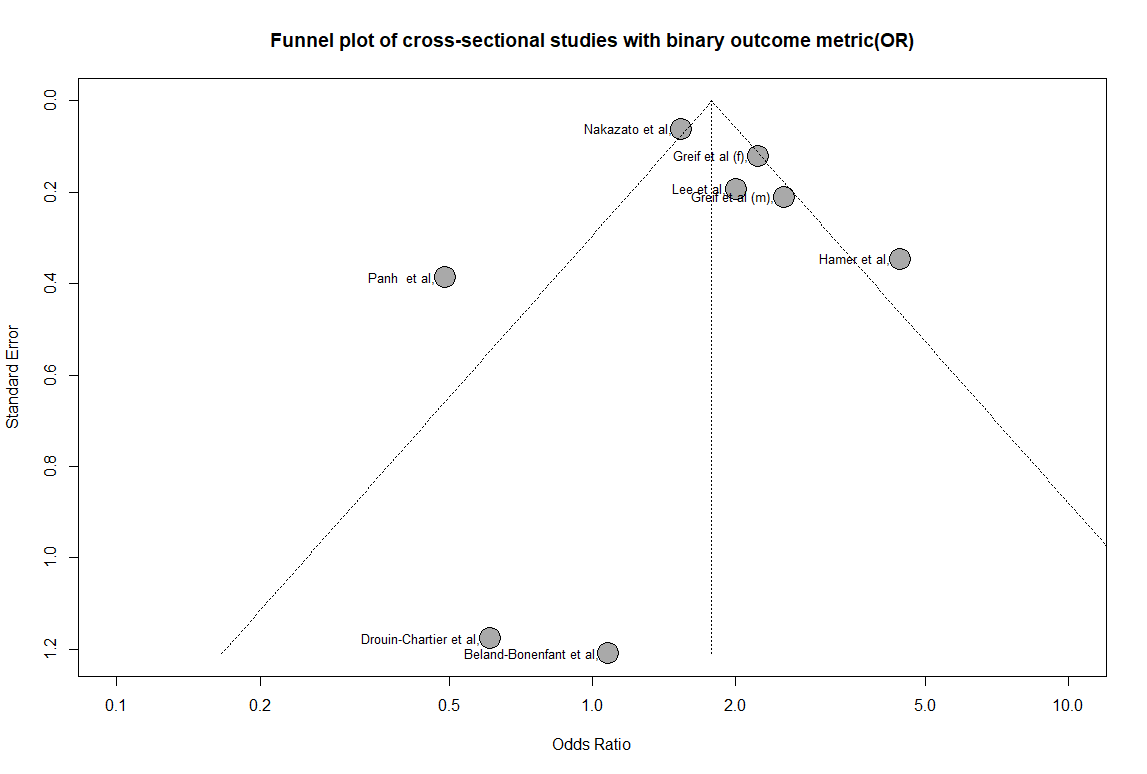
**

Figure S17:

**
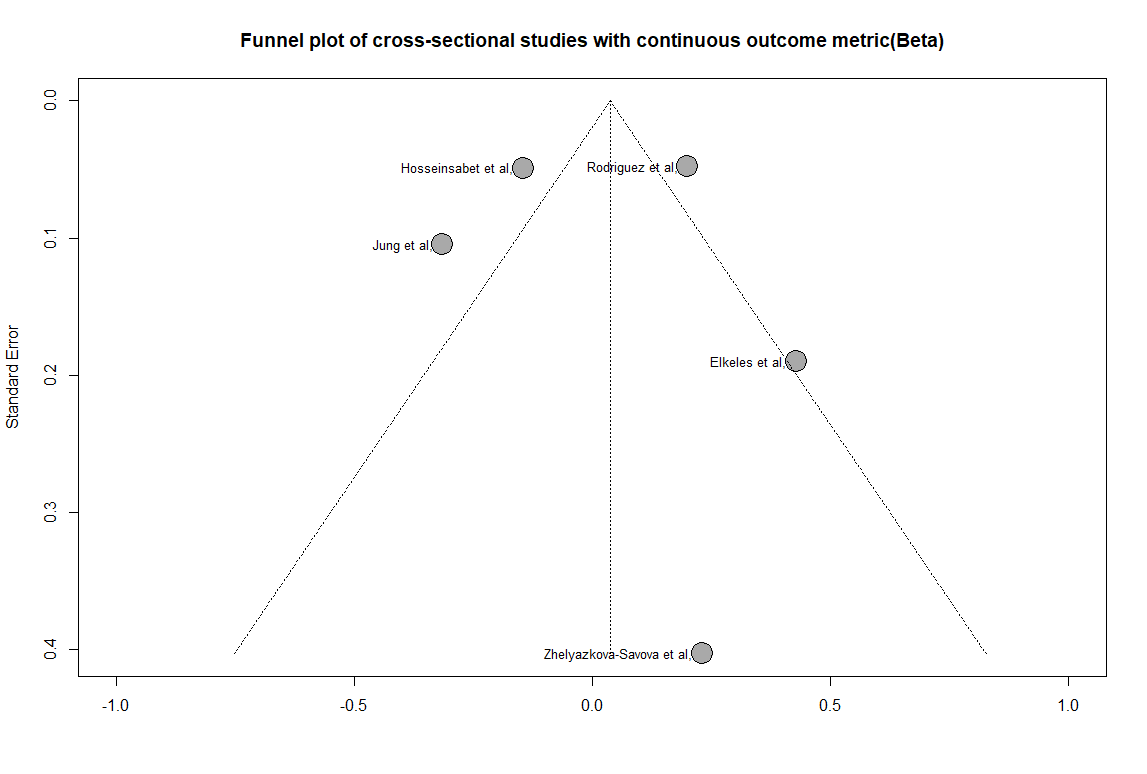
**

Figure S18:

**
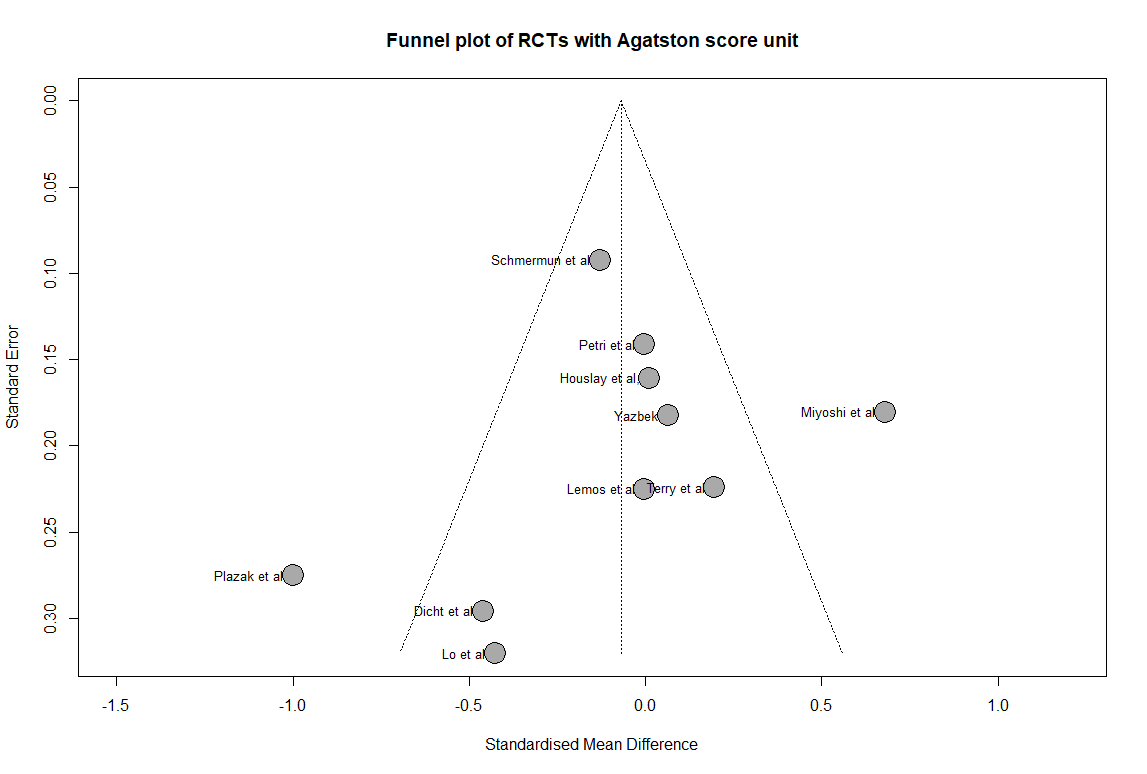
**

**Figure S19- S20:** Funnel plot and contour enhance funnel plot

**
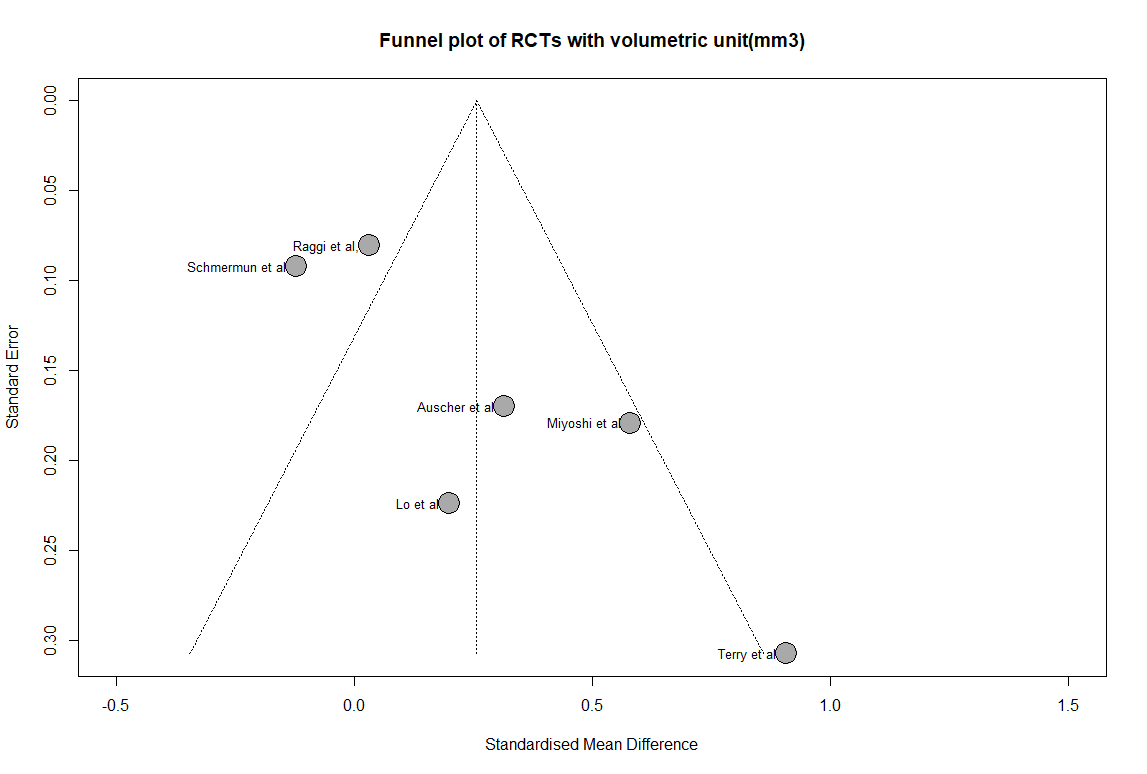
** **
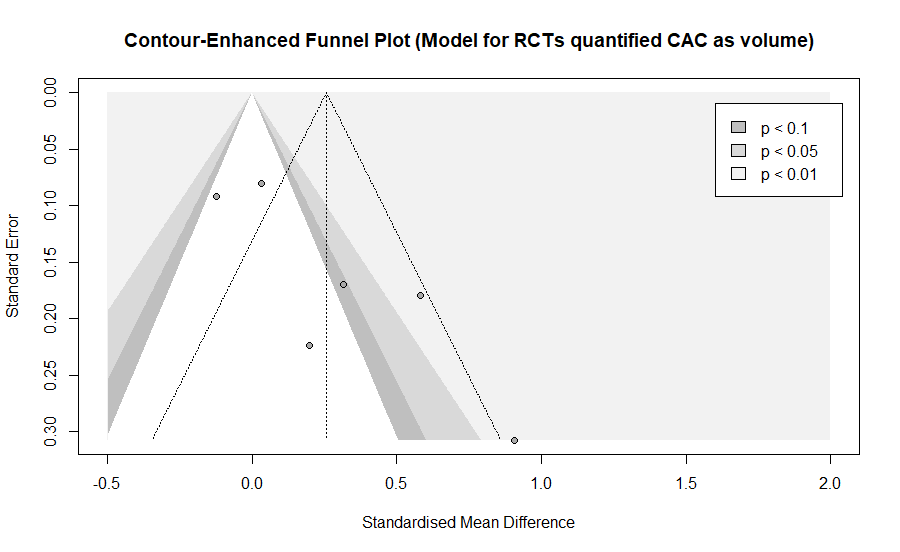
**

**Egger’s test**

Table S10: Egger’s regression test for the presence of asymmetry in the funnel plot.

| Model,  Effect size | Egger’s  Intercept | 95% Confidence interval | t-value | p-value* |
| --- | --- | --- | --- | --- |
| Cohort  OR | -0.73 | -9.77, 8.30 | -0.16 | 0.89 |
| Cohort  Beta | -1.70 | -3.60, 0.19 | -1.76 | 0.22 |
| Cross-sectional  OR | 0.31 | -2.00, 2.63 | 0.26 | 0.80 |
| Cross-sectional  Beta | 0.22 | -5.67, 6.12 | 0.075 | 0.94 |
| RCT  Agatston score | -0.84 | -4.22, 2.54 | -0.49 | 0.64 |
| RCT  Volume | 3.78 | 1.41, 6.16 | 3.12 | 0.03 |

OR: Odds Ratio, SMD: Standardized mean difference, *: The two-tailed P value.

- - - 1. **Synthesis without meta-analysis (SWiM)**

Table S11: Synthesis without meta-analysis (SWiM) of cohort studies and the summary of effect estimates.

|  | **Source** | **N** | **Follow-up, y** | **QAR*** | **Calcification Quantification** | **Outcome metric and description** | **Effect summary** | **Effect direction plot^1^** | **Reason for exclusion from meta-analysis** |
| --- | --- | --- | --- | --- | --- | --- | --- | --- | --- |
| **Cohorts**  **Dichotomous outcome** | | | | | | | | | |
|  | Pechlivanis et al , 2021 (Germany) | 3157 | 5.1 | 8/9 | AS | Binary, CAC progression as either rapid or expected/slow | OR>1  95%CI=significant |  | Different outcome description. Clinical and methodological diversity. Limited evidence for a pre-specified comparison. |
| **Cohorts**  **Continuous outcome** | | | | | | | | | |
| 1. 2. | Budoff et al, 2005 (USA) | 163 | 2.3 | 7/9 | AS | Continuous,  Median annualized change in CAC | Beta<0  95%CI=significant |  | Different outcome description. Limited evidence for a pre-specified comparison^2^. |
| 1. 6. | Hoffmann et al, 2010 (Germany) | 63 | 2.08 | 8/9 | mm3 | Continuous,  Growth of calcified plaques quantified by CTA | Beta>0  95%CI=non-significant |  | Different outcome and exposure description. Methodological diversity. Limited evidence for a pre-specified comparison. |
| 1. 8. | Zeb et al, 2013 (USA) | 100 | 1.1 | 7/9 | mm3 | Continuous,  Change in CAC quantified by CTA | Beta>0  95%CI=non-significant |  | Different outcome description. Limited evidence for a pre-specified comparison. |
| 1. 15. | Smit et al, 2020 (5 European countries) | 202 | 6.2 | 8/9 | mm3 | Continuous,  annual change in CAC quantified by CTA | Beta>0  95%CI= significant |  | Different outcome description. Methodological diversity,  Limited evidence for a pre-specified comparison. |

*QAR: Quality assessment results, CAC= Coronary artery calcification, CTA=Computed Tomography Angiography.

1: Effect direction plot; direction: the upward arrow (increase in calcification), the downward arrow (decrease in calcification). Statistical significance: the solid arrow (significant), the plane arrow (non-significance). Sample size: large arrow (n≥200), small arrow(n<200). ), Table show results according to SWiM guideline (4), and the direction of the effect plot (5).

2: Authors have been contacted by email however, limited evidence available for a pre-specified comparison.

Table S12: Synthesis without meta-analysis (SWiM) of cross-sectional studies and the summary of effect estimates.

|  | **Source** | **N** | **QAR*** | **Calcification Quantification** | **Outcome metric and description** | **Effect estimate’s summary** | **Effect direction plot^1^** | **Reason for exclusion from meta-analysis** |
| --- | --- | --- | --- | --- | --- | --- | --- | --- |
| **Cross-sectional**  **Dichotomous outcome** | | | | | | | | |
| 1 | Jeon et al, 2010 (South Korea) | 436 | 8/9 | AS | Binary, CAC score >100 | OR>1  95%CI= non- significant |  | Different outcome description. |
| 2 | Shikada et al , 2015 (Japan) | 201 | 7/9 | AS | Binary, CAC score >238 | OR>1  95%CI= significant |  | Different outcome description. |
| **Cross-sectional**  **Continuous outcome** | | | | | | | | |
| 3 | Nguyen et al, 2007 (Belgium) | 281 | 8/9 | Calcium mass score | Continuous, Calcium mass score | Beta>0  95%CI= significant |  | Different quantification method |
| 4 | Cheng et al, 2010 (USA) | 823 | 7/9 | Count | Continuous, Absolute calcified plaque count (>75% calcified) | Beta>0  95%CI= significant |  | Different outcome unit |

*QAR: Quality assessment results, CAC= Coronary artery calcification,

1: Effect summary; direction: upward arrow (increase in calcification), downward arrow (decrease in calcification). Statistical significance: solid arrow (significant), plane arrow (non-significance), Sample size: large arrow (n≥200), small arrow (n<200), Table was designed according to SWiM guideline (4), and direction of effect plot (5).

- - - 1. **Grading of Recommendations Assessment, Development and Evaluation (GRADE)**

Table S13 : Grading of Recommendations Assessment, Development and Evaluation (GRADE) for the main outcomes of synthesis with and without meta-analysis

| **Index** | | **Outcome** | **Certainty assessment criteria** | | **№ of patients** | | **Effect** | | **Certainty of evidence ^8^** |  |
| --- | --- | --- | --- | --- | --- | --- | --- | --- | --- | --- |
| № of row | № of included studies |  | **Downgrading factors:**   1. Risk of Bias 2. Inconsistency 3. Indirectness 4. Imprecision | **Upgrading factors:**   1. Dose-response gradient 2. Large effect 3. No plausible confounding | Total number of patients | | Effect estimate* [measure of precision] | Summary** |  |  |
| **Observational (cohort) studies; initial rate LOW** | | | | | | | | | |  |
| **Binary effect metric (OR):** | | | | | | | | | |  |
| 1. 7. | 1 | Rapid Progression of CAC | 1. Not Serious 2. Not serious 3. Not Serious 4. Not serious | 1. No 2. No 3. No | Baseline  3,157 | Follow-up  3,157 | OR>1  CI=significant |  | ⨁⨁◯◯ Low |  |
| 1. 8. | 4 | Progression of CAC | 1. Not serious 2. Serious 3. Not serious 4. Serious | 1. No 2. No 3. No | Baseline  2,053 | Follow-up  2,049 | Pooled OR= 1.10  [95%CI= 0.57; 2.12] |  | ⨁◯◯◯  Very Low |  |
| **Continuous effect metric (Beta):** | | | | | | | | | |  |
| 3. | 3 | Agatston score | 1. Not serious 2. Not Serious 3. Not serious 4. Not Serious | 1. No 2. No 3. No | Baseline  1,304 | Follow-up  781 | Pooled ẞ = 0.11,  [95%CI= 0.05; 0.16] |  | ⨁⨁◯◯ Low |  |
| 4. | 1 | Volume (mm3) | 1. Not Serious 2. Not serious 3. Serious 4. Not serious | 1. No 2. No 3. No | Baseline  202 | Follow-up  202 | Beta>0  CI= significant |  | ⨁◯◯◯  Very Low |  |
| 5. | 1 | Agatston score | 1. Serious 2. Not serious 3. Serious 4. Serious | 1. No 2. Yes 3. No | Baseline  163 | Follow-up  163 | Beta<0  CI=significant |  | ⨁⨁◯◯ Low |  |
| 6. | 1 | Volume (mm3) | 1. Serious 2. Not serious 3. Serious 4. Serious | 1. No 2. Yes 3. No | Baseline  100 | Follow-up  100 | Beta>0  CI=non-significant |  | ⨁⨁◯◯ Low |  |
| 7. | 1 | Volume (mm3) | 1. Not serious 2. Not serious 3. Serious 4. Not serious | 1. Yes 2. No 3. No | Baseline  63 | Follow-up  63 | Beta>0  CI=non-significant |  | ⨁⨁◯◯ Low |  |
| **Observational (cross-sectional) studies; initial rate LOW** | | | | | | | | | |  |
| **Binary effect metric (OR):** | | | | | | | | | |  |
| 8. | 7 | CAC>0  (presence of CAC) | 1. Not serious 2. Not serious 3. Not serious 4. Not serious | 1. No 2. No 3. No | 9,731 | | Pooled OR = 2.11,  [95%CI= 1.61; 2.78] |  | ⨁⨁◯◯ Low |  |
| 9. | 1 | CAC>100  (Agatston score) | 1. Not serious 2. Not serious 3. Serious 4. Serious | 1. No 2. No 3. No | 436 | | OR>1  [non- significant] |  | ⨁◯◯◯ Very Low |  |
| 10. | 1 | CAC>238  (Agatston score) | 1. Serious 2. Not serious 3. Serious 4. Not serious | 1. No 2. No 3. No | 201 | | OR>1  CI=significant |  | ⨁◯◯◯  Very Low |  |
| **Continuous effect metric (Beta):** | | | | | | | | | |  |
| 11. | 5 | Agatston score | 1. Not serious 2. Serious 3. Not serious 4. Serious | 1. No 2. No 3. No | 1,048 | | Pooled ẞ = 0.04,  [95%CI= -0.24; 0.31] |  | ⨁◯◯◯  Very Low |  |
| 12. | 1 | Count (number of absolute calcified plaques) | 1. Serious 2. Not serious 3. Serious 4. Not serious | 1. No 2. No 3. No | 823 | | Beta>0  CI=significant |  | ⨁◯◯◯  Very Low |  |
| 13. | 1 | Calcium mass score | 1. Not Serious 2. Not serious 3. Serious 4. Not serious | 1. No 2. No 3. No | 281 | | Beta>0  CI=significant |  | ⨁◯◯◯  Very Low |  |
| **Randomized Controlled Trials; initial rate HIGH** | | | | | | | | | |  |
| **Standardized mean difference (SMD): CAC quantified in volume (mm^3^).** | | | | | | | | | |  |
| 14. | 6 | Volume (mm3) | 1. Not serious 2. Serious*** 3. Not serious 4. Not serious | 1. No 2. No 3. Yes | Intervention arm, baseline:  740 | Control arm, baseline:  743 | Pooled SMD=0.26,  [95%CI= -0.11; 0.63] |  | ⨁⨁⨁⨁ High |  |
| **Standardized mean difference (SMD): CAC quantified in Agatston score.** | | | | | | | | | |  |
| 15. | 8 | Agatston score | 1. Not serious 2. Not serious 3. Not serious 4. Not serious | 1. No 2. No 3. Yes | Intervention arm, baseline:  592 | Control arm, baseline:  600 | Pooled SMD= -0.06  [95%CI= -0.19; 0.06] |  | ⨁⨁⨁⨁ High |  |

The GRADE approach suggests rating the quality of evidence for each outcome; RCTs start with a high rating, and observational studies with a low rating. Rating is modified downward or upward according to the 7 criteria:

1. The risk of bias was assessed according to the results of the quality assessment of the study’s methodology. Cochrane tool for risk of bias assessment has been used for RCTs and the Newcastle Ottawa checklist was used for observational studies. Levels: Not serious, serious (-1), very serious (-2).
2. Inconsistency of evidence was assessed based on whether there is unexplained heterogeneity identified by analysis of point estimates and confidence interval overlap, statistical test (chi-square), and statistical estimates (I2). Levels: Not serious, serious (-1), very serious (-2).
3. Indirectness of evidence was assessed based on whether there was any substantial difference between the identified evidence and the original question of the review regarding patients, interventions, comparisons, and outcomes to the extent that these differences would question the obtained effect estimate related to the review or guideline question PICO: population, intervention, comparison, and outcome. Levels: Not serious, serious (-1), very serious (-2).
4. Imprecision of evidence was assessed based on the interpretation of the limits of the confidence interval, determine whether the optimal information size is met. Levels: Not serious, serious (-1), very serious (-2).
5. Dose-response gradient was assessed based on whether studies represented clear evidence of the dose-response gradient or not. Levels: Yes, no.
6. Large effect was assessed based on Cohen interpretation. An explicit description of the magnitude of the effect is considered as large. Also based on whether a large effect or association is drawn based on well-done observational studies without important risk of bias or other limitations is present and the time frame of exposure necessary to achieve the effect. Levels: Yes, no.
7. Addressing plausible confounding was assessed whether the studies provide evidence of all plausible confounders or biases in favour of detecting an effect or association when the latter is not detected. Explicit description of the mechanism for which confounders or biases may reduce or increase the observed effect or association. Levels: Yes, no.

*Effect estimates for outcomes synthesized with meta-analysis is the pooled effect in the corresponding 95%CI after excluding the outliers and influential cases. For outcome synthesized without meta-analysis is the effect estimate according to the SWiM (synthesis without meta-analysis) (4) results.

**Effect summary; direction: upward arrow (increase in calcification), a downward arrow (decrease in calcification). Statistical significance: solid arrow (significant), a plane arrow (non-significance). Sample size: large arrow (n≥200), a small arrow(n<200), summarized by using the effect direction plots (5).

CI: Confidence Interval, OR: Odds Ratio, ẞ: Beta coefficient.

***Small study effect and publication bias observed

1. **List of a number of potentially relevant studies not included in the systematic review, along with the reasons for exclusion.**

Table S14: List of a number of potentially relevant studies not included in the systematic review, along with the reasons for exclusion.

| Number | REFERENCE | Reason for exclusion |
| --- | --- | --- |
|  | Lee SE, Sung JM, Andreini D, Budoff MJ, Cademartiri F, Chinnaiyan K, Choi JH, Chun EJ, Conte E, Gottlieb I, Hadamitzky M. Differential association between the progression of coronary artery calcium score and coronary plaque volume progression according to statins: the Progression of AtheRosclerotic PlAque DetermIned by Computed TomoGraphic Angiography Imaging (PARADIGM) study. European Heart Journal-Cardiovascular Imaging. 2019 Nov 1;20(11):1307-14. | No effect size for required association |
|  | Karpouzas GA, Ormseth SR, Hernandez E, Budoff MJ. The impact of statins on coronary atherosclerosis progression and long-term cardiovascular disease risk in rheumatoid arthritis. Rheumatology. 2021 Aug 9. | Post hoc analysis |
|  | Schmid M, Achenbach S, Ropers D, Komatsu S, Ropers U, Daniel WG, Pflederer T. Assessment of changes in non-calcified atherosclerotic plaque volume in the left main and left anterior descending coronary arteries over time by 64-slice computed tomography. The American journal of cardiology. 2008 Mar 1;101(5):579-84. | No calcification as the outcome |
|  | Dykun I, Lehmann N, Kälsch H, Möhlenkamp S, Moebus S, Budde T, Seibel R, Grönemeyer D, Jöckel KH, Erbel R, Mahabadi AA. Statin medication enhances progression of coronary artery calcification: the Heinz Nixdorf recall study. Journal of the American College of Cardiology. 2016 Nov 8;68(19):2123-5. | Letter: other types of publication |
|  | Al Rifai M, Blaha MJ, Patel J, Xiaoming J, Cainzos-Achirica M, Greenland P, Budoff M, Yeboah J, Nasir K, Al-Mallah MH, Virani SS. Coronary artery calcification, statin use and long-term risk of atherosclerotic cardiovascular disease events (from the multi-ethnic study of atherosclerosis). The American Journal of Cardiology. 2020 Mar 15;125(6):835-9. | No effect estimate for required association |
|  | Shin S, Park HB, Chang HJ, Arsanjani R, Min JK, Kim YJ, Lee BK, Choi JH, Hong GR, Chung N. Impact of intensive LDL cholesterol lowering on coronary artery atherosclerosis progression: a serial CT angiography study. JACC: Cardiovascular Imaging. 2017 Apr;10(4):437-46. | No effect estimate for required association |
|  | Ceponiene I, Nakanishi R, Osawa K, Kanisawa M, Nezarat N, Rahmani S, Kissel K, Kim M, Jayawardena E, Broersen A, Kitslaar P. Coronary artery calcium progression is associated with coronary plaque volume progression: results from a quantitative semiautomated coronary artery plaque analysis. JACC: Cardiovascular Imaging. 2018 Dec;11(12):1785-94. | No effect estimate for required association |
|  | Soeda T, Uemura S, Okayama S, Kawakami R, Sugawara Y, Nakagawa H, Matsumoto T, Sung JH, Nishida T, Senoo A, Somekawa S. Intensive Lipid-Lowering Therapy With Rosuvastatin Stabilizes Lipid-Rich Coronary Plaques–Evaluation Using Dual-Source Computed Tomography–. Circulation Journal. 2011;75(11):2621-7. | No effect estimate for required association |
|  | Sandfort V, Lai S, Ahlman MA, Mallek M, Liu S, Sibley CT, Turkbey EB, Lima JA, Bluemke DA. Obesity is associated with progression of atherosclerosis during statin treatment. Journal of the American Heart Association. 2016 Jul 13;5(7):e003621. | No effect estimate for required association |
|  | Wu YW, Kao HL, Huang CL, Chen MF, Lin LY, Wang YC, Lin YH, Lin HJ, Tzen KY, Yen RF, Chi YC. The effects of 3-month atorvastatin therapy on arterial inflammation, calcification, abdominal adipose tissue and circulating biomarkers. European journal of nuclear medicine and molecular imaging. 2012 Mar;39(3):399-407. | No value of CAC for control group |
|  | Noguchi T, Tanaka A, Kawasaki T, Goto Y, Morita Y, Asaumi Y, Nakao K, Fujiwara R, Nishimura K, Miyamoto Y, Ishihara M. Effect of intensive statin therapy on coronary high-intensity plaques detected by noncontrast T1-weighted imaging: the AQUAMARINE pilot study. Journal of the American College of Cardiology. 2015 Jul 21;66(3):245-56. | MRI (no CT scan) |
|  | Svanteson M, Rollefstad S, Kløw NE, Hisdal J, Ikdahl E, Sexton J, Haig Y, Semb AG. Effects of long-term statin-treatment on coronary atherosclerosis in patients with inflammatory joint diseases. PloS one. 2019 Dec 12;14(12):e0226479. | No effect estimate reported for the association |
|  | Longenecker CT, Sattar A, Gilkeson R, McComsey GA. Rosuvastatin slows progression of subclinical atherosclerosis in patients with treated HIV infection. AIDS (London, England). 2016 Sep 10;30(14):2195. | No CAC value for all participants |
|  | Henein M, Granåsen G, Wiklund U, Schmermund A, Guerci A, Erbel R, Raggi P. High dose and long-term statin therapy accelerate coronary artery calcification. International journal of cardiology. 2015 Apr 1;184:581-6. | Pooled analysis of 2 studies. One is already been included. Another has been excluded |
|  | Burgstahler C, Reimann A, Beck T, Kuettner A, Baumann D, Heuschmid M, Brodoefel H, Claussen CD, Kopp AF, Schroeder S. Influence of a lipid-lowering therapy on calcified and noncalcified coronary plaques monitored by multislice detector computed tomography: results of the New Age II Pilot Study. Investigative radiology. 2007 Mar 1;42(3):189-95. | Effect of ASA plus statin |
|  | Saremi A, Bahn G, Reaven PD, VADT Investigators. Progression of vascular calcification is increased with statin use in the Veterans Affairs Diabetes Trial (VADT). Diabetes care. 2012 Nov 1;35(11):2390-2. | Brief report: other types of publication |
|  | Shimojima M, Kawashiri MA, Nitta Y, Yoshida T, Katsuda S, Kaku B, Taguchi T, Hasegawa A, Konno T, Hayashi K, Yamagishi M. Rapid changes in plaque composition and morphology after intensive lipid lowering therapy: study with serial coronary CT angiography. American journal of cardiovascular disease. 2012;2(2):84. | No effect estimate reported for the association |
|  | Lipinski J, Margevicius S, Schluchter MD, Wilson DL, McComsey GA, Longenecker CT. Statin effect on coronary calcium distribution, mass and volume scores and associations with immune activation among HIV+ persons on antiretroviral therapy. | Short communication: other types of publication, No effect estimate reported for the association |
|  | Mizuno K, Nakamura H, Ohashi Y, Kaburagi T, Kitabatake A, Tochihara T, Hosoda S, JUST Study Investigators. A randomized, open-label, comparative study of simvastatin plus diet versus diet alone on angiographic retardation of coronary atherosclerosis in adult Japanese patients: Japanese utilization of simvastatin therapy (JUST) study. Clinical therapeutics. 2004 Jun 1;26(6):878-88. | No CAC value was measured before and after the statin intervention |
|  | Lee SE, Chang HJ, Sung JM, Park HB, Heo R, Rizvi A, Lin FY, Kumar A, Hadamitzky M, Kim YJ, Conte E. Effects of statins on coronary atherosclerotic plaques: the PARADIGM study. JACC: Cardiovascular Imaging. 2018 Oct;11(10):1475-84. | No Beta or Odds ratio estimated for required association |
|  | Arad Y, Spadaro LA, Roth M, Newstein D, Guerci AD. Treatment of asymptomatic adults with elevated coronary calcium scores with atorvastatin, vitamin C, and vitamin E: the St. Francis Heart Study randomized clinical trial. Journal of the American College of Cardiology. 2005 Jul 5;46(1):166-72. | Effect of Vit E and C plus statin |
|  | Sjölander M, Carlberg B, Norberg M, Näslund U, Ng N. Prescription of Lipid-Lowering and Antihypertensive Drugs Following Pictorial Information About Subclinical Atherosclerosis: A Secondary Outcome of a Randomized Clinical Trial. JAMA network open. 2021 Aug 2;4(8):e2121683-. | No effect estimate for required association |
|  | Zafeiropoulos S, Farmakis I, Kartas A, Arvanitaki A, Pagiantza A, Boulmpou A, Tampaki A, Kosmidis D, Nevras V, Markidis E, Papadimitriou I. Reinforcing adherence to lipid-lowering therapy after an acute coronary syndrome: a pragmatic randomized controlled trial. Atherosclerosis. 2021 Apr 1;323:37-43. | No CAC value before and after statins |
|  | Van Rosendael AR, Van Den Hoogen IJ, Gianni U, Ma X, Tantawy SW, Bax AM, Lu Y, Andreini D, Al-Mallah MH, Budoff MJ, Cademartiri F. Association of statin treatment with progression of coronary atherosclerotic plaque composition. JAMA cardiology. 2021 Nov 1;6(11):1257-66. | Full text not available, in spite of contacting the authors |
|  | Mortensen MB, Falk E, Li D, Nasir K, Blaha MJ, Sandfort V, Rodriguez CJ, Ouyang P, Budoff M. Statin trials, cardiovascular events, and coronary artery calcification: implications for a trial-based approach to statin therapy in MESA. JACC: Cardiovascular Imaging. 2018 Feb;11(2 Part 1):221-30. | No effect estimate for required association |
|  | Yu W, Ji H, Tan Q. Evaluation of the Efficacy of Statins in the Treatment of Coronary Artery Plaque Using Dual-Source Spiral Computed Tomography Image Features under Deep Learning. Scientific Programming. 2022 Jan 7;2022. | No effect estimate for required association |
|  | Miname MH, Bittencourt MS, Moraes SR, Alves RI, Silva PR, Jannes CE, Pereira AC, Krieger JE, Nasir K, Santos RD. Coronary artery calcium and cardiovascular events in patients with familial hypercholesterolemia receiving standard lipid-lowering therapy. JACC: Cardiovascular Imaging. 2019 Sep;12(9):1797-804. | No effect estimate for required association |
|  | Nicholls SJ, Tuzcu EM, Wolski K, Sipahi I, Schoenhagen P, Crowe T, Kapadia SR, Hazen SL, Nissen SE. Coronary artery calcification and changes in atheroma burden in response to established medical therapies. Journal of the American College of Cardiology. 2007 Jan 16;49(2):263-70. | No effect estimate for required association |
|  | Callister TQ, Raggi P, Cooil B, Lippolis NJ, Russo DJ. Effect of HMG-CoA reductase inhibitors on coronary artery disease as assessed by electron-beam computed tomography. New England Journal of Medicine. 1998 Dec 31;339(27):1972-8. | No effect estimate for required association |
|  | Tenenbaum A, Shemesh J, Koren-Morag N, Fisman EZ, Adler Y, Goldenberg I, Tanne D, Hay I, Schwammenthal E, Motro M. Long-term changes in serum cholesterol level does not influence the progression of coronary calcification. International journal of cardiology. 2011 Jul 15;150(2):130-4. | Lipid lowering therapy in general, No effect estimate for required association |
|  | Svanteson M, Rollefstad S, Kløw NE, Hisdal J, Ikdahl E, Sexton J, Haig Y, Semb AG. Effects of long-term statin-treatment on coronary atherosclerosis in patients with inflammatory joint diseases. PloS one. 2019 Dec 12;14(12):e0226479. | No control group. No randomization |
|  | Achenbach S, Ropers D, Pohle K, Leber A, Thilo C, Knez A, Menendez T, Maeffert R, Kusus M, Regenfus M, Bickel A. Influence of lipid-lowering therapy on the progression of coronary artery calcification: a prospective evaluation. Circulation. 2002 Aug 27;106(9):1077-82. | No randomization |
|  | Mok CC, Wong CK, To CH, Lai JP, Lam CS. Effects of rosuvastatin on vascular biomarkers and carotid atherosclerosis in lupus: A randomized, double‐blind, placebo‐controlled trial. Arthritis Care & Research. 2011 Jun;63(6):875-83. | Carotid artery |
|  | Engbers EM, Timmer JR, Mouden M, Knollema S, Jager PL, Ottervanger JP. Changes in cardiovascular medication after coronary artery calcium scanning and normal single photon emission computed tomography myocardial perfusion imaging in symptomatic patients. American heart journal. 2017 Apr 1;186:56-62. | No effect estimate for required association |
|  | Goh VK, Lau CP, Mohlenkamp S, Rumberger JA, Achenbach S, Budoff MJ. Outcome of coronary plaque burden: a 10-year follow-up of aggressive medical management. Cardiovascular ultrasound. 2010 Dec;8(1):1-1. | No effect estimate for required association |
|  | Forbat SM, Naoumova RP, Sidhu PS, Neuwirth C, MacMahon M, Thompson GR, Underwood SR. The effect of cholesterol reduction with fluvastatin on aortic compliance, coronary calcification and carotid intimal-medial thickness: a pilot study. Journal of Cardiovascular Risk. 1998 Feb;5(1):1-0. | MRI, no base line and follow up CAC, no control |
|  | Budoff MJ, Lane KL, Bakhsheshi H, Mao S, Grassmann BO, Friedman BC, Brundage BH. Rates of progression of coronary calcium by electron beam tomography. The American journal of cardiology. 2000 Jul 1;86(1):8-11. | No effect estimate for required association |

1. **References:**

1. Wells G, Shea B, O’Connell D, Peterson J, Welch V, Losos M, et al. Newcastle-Ottawa quality assessment scale cohort studies. University of Ottawa. 2014.

2. Wells GA, Shea B, O’Connell D, Peterson J, Welch V, Losos M, et al. The Newcastle-Ottawa Scale (NOS) for assessing the quality of nonrandomised studies in meta-analyses. Oxford; 2000.

3. Higgins JPT, Altman DG. Assessing risk of bias in included studies. 2008.

4. Campbell M, McKenzie JE, Sowden A, Katikireddi SV, Brennan SE, Ellis S, et al. Synthesis without meta-analysis (SWiM) in systematic reviews: reporting guideline. bmj. 2020;368.

5. Thomson HJ, Thomas S. The effect direction plot: visual display of non‐standardised effects across multiple outcome domains. Research synthesis methods. 2013;4(1):95-101.
